# Conjugated Multiblock Copolymers and Microcracked Gold Electrodes Applied for the Intrinsically Stretchable Field-Effect Transistor

**DOI:** 10.1021/acsami.5c00047

**Published:** 2025-03-27

**Authors:** Yu-Chun Huang, Shuto Yamamoto, Jung-Yao Chen, Chun-Jen Su, U-Ser Jeng, Tomoya Higashihara, Yan-Cheng Lin

**Affiliations:** † Department of Chemical Engineering, National Cheng Kung University, Tainan 70101, Taiwan; ‡ Department of Organic Materials Science, Graduate School of Organic Materials Science, Yamagata University, 4-3-16 Jonan, Yonezawa, Yamagata 990-0021, Japan; § Department of Photonics, National Cheng Kung University, Tainan City 70101, Taiwan; ∥ National Synchrotron Radiation Research Center, Hsinchu 300092, Taiwan; ⊥ Department of Chemical Engineering & College of Semiconductor Research, National Tsing Hua University, Hsinchu 300044, Taiwan; # Advanced Research Center for Green Materials Science and Technology, National Taiwan University, Taipei 10617, Taiwan

**Keywords:** block copolymers, microcracked gold, N-type conjugated polymers, organic field-effect transistor, stretchable electronics

## Abstract

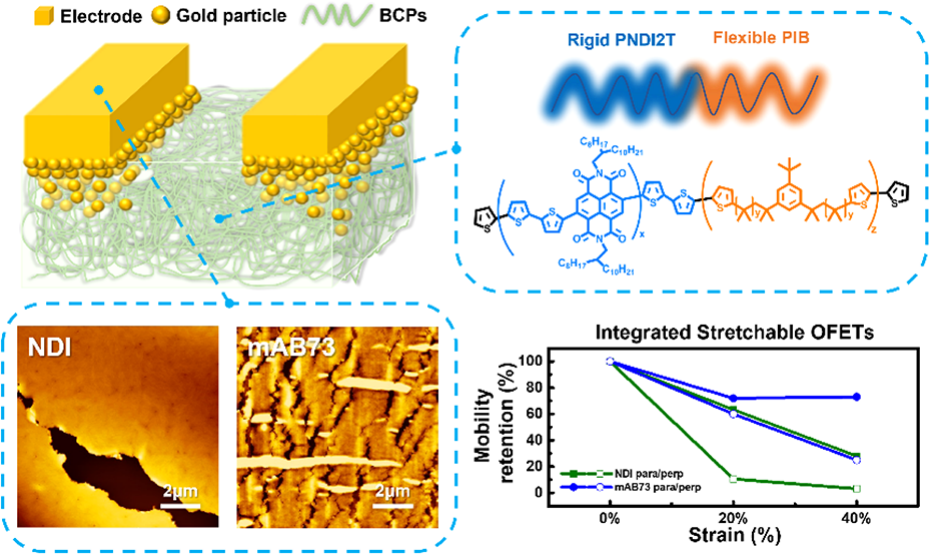

The rise of flexible electronic devices has led to extensive research into conjugated polymer structural engineering. Integrating polymer channels and contact electrodes, warranting high stretchability, is still critical, and the microcracked gold technique provides a potential strategy to integrate them. Conjugated block copolymers have gained significant attention due to their high flexibility, allowing for tailored polymer structures to meet the specific requirements of different device characteristics. In this study, novel N-type multiblock copolymers (multi-BCPs) composed of rigid poly(naphthalene diimide-*alt*-bithiophene) and flexible polyisobutylene segments were successfully synthesized as polymer semiconductors for the first time. The materials are named based on the weight fraction of soft segments: NDI (0 wt %), mAB73 (27 wt %), and mAB60 (40 wt %). The study explores the mechanical properties, crystallinity, and electrical performance of flexible multi-BCPs. The results show that introducing soft segments significantly enhances stretchability, with crack-onset strains beyond 100% because of their low elastic moduli of 40–50 MPa. Furthermore, the OFET device of mAB73 achieves unchanged mobility under 100% strain, outperforming mAB60 due to excessive polyisobutylene blocks. At the end of this study, an integrated stretchable device with high stretchability is fulfilled by utilizing the microcracked gold technique to combine the multi-BCP channels and contact electrodes. The integrated device can be applied to biomedical electronics without toxic or corrosive electrode materials. The influencing factors, including contact resistance, channel charge mobility, and electrode resistance, are systematically studied to investigate the integrated device’s mobility–stretchability relationship. The results indicate that the contact resistance between the multi-BCP channels and contact electrodes is essential to the device’s performance. Among these, mAB73, containing soft segments, exhibits more stability than NDI due to the microcracked gold electrodes with infiltrated gold nanoparticles in the rubbery channel surface. Appropriately incorporating soft segments significantly enhances mobility retention under tensile strains, highlighting the potential of multi-BCP designs in stretchable electronic applications.

## Introduction

With the rapid advancement of the Internet of Things (IoT), wearable electronic devices have become an increasingly significant area of focus. These devices are extensively utilized in applications such as electronic skin, flexible displays, biosensors, and soft robotics.^1−5^ A core element of these technologies is flexible electronics, which are distinguished by their ability to maintain favorable electrical properties under various mechanical stresses, such as bending, stretching, and compression. The development of flexible electronics has paved the way for innovative devices that respond to mechanical deformation without compromising their performance. Flexible electronic devices are created through different fabrication processes and serve multiple purposes. Some notable examples include organic field-effect transistors (OFETs),^6,7^ photovoltaics (OPVs),^8,9^ light-emitting diodes (OLEDs),^10−12^ and thermoelectrics (OTEs).^13,14^ A key material in these applications is conjugated polymers, which have garnered significant attention due to their exceptional mechanical properties, solution processability, flexibility, and low cost. The versatility of conjugated polymers has sparked widespread interest among researchers in chemistry, physics, and engineering, particularly in their application to flexible electronics. Achieving a balance between high charge mobility, stretchability, and cycling stability under mechanical deformation is critical for the development of high-performance stretchable polymer semiconductors. Conjugated polymers can be engineered chemically, physically, and structurally to improve these attributes. However, these materials have a complex relationship among crystallinity, stretchability, and charge transport. Researchers must carefully navigate the trade-offs between these properties to achieve optimal performance, making it a critical challenge in the field.

Among flexible electronic devices, OFETs stand out as some of the most widely utilized. These devices typically comprise crucial components, such as electrodes, polymer semiconductors, and dielectric layers. Both the electrodes and dielectric layers need to maintain elasticity to ensure flexibility. Materials commonly used in this context include poly(3,4-ethylenedioxythiophene):poly(styrenesulfonate) (PEDOT:PSS),^15,16^ single-walled carbon nanotubes (SWCNTs),^17,18^ polydimethylsiloxane (PDMS),^19−21^ and styrene-ethylene-butylene-styrene rubber (SEBS).^22,23^ These materials are selected for their ability to retain stable conductivity and capacitance even when subjected to mechanical stress. Maintaining efficient charge transport while preserving stretchability is paramount in the design of stretchable polymer semiconductors.^24−26^ Structural engineering plays a crucial role in enhancing these properties. One approach involves modifying the side chains of polymers with long alkyl or branched alkyl groups, which can significantly improve the stretchability of polymer semiconductors.^27,28^ However, while these modifications increase flexibility, they often lead to a reduction in polymer crystallinity, which can decrease charge carrier mobility. In addition to side-chain modifications, researchers have explored introducing conjugated break spaces (CBS) into the polymer backbone to disrupt excessive crystallinity.^29,30^ This modification allows the polymer to retain its mechanical and electrical performance despite the reduction in crystallinity. Both side-chain and backbone modifications have shown promise in improving the mechanical properties of polymer semiconductors, but each approach comes with its challenges. Block copolymers (BCPs) have recently emerged as compelling solutions to address these limitations. BCPs have shown great potential in fabricating polymer semiconductors for OFETs due to their ability to incorporate different block structures that enhance stretchability while minimizing the negative impact on charge transport.^31−35^ Sugiyama et al. developed the first p-type multiblock conjugated BCPs comprising poly(ε-caprolactone) (PCL) and diketopyrrolopyrrole-based conjugated polymers, showing a high crack-onset strain >100%.^36^ However, these BCPs are p-type, and their mobility variations under strain have not been revealed. Peng et al. utilized conjugated BCPs composed of various ratios of poly(3-butylthiophene) (P3HT) and poly(3-hexylselenophene) (P3HS), which self-assembled to form cocrystals. This approach enabled high charge mobility performance without thermal annealing.^37^ In our previous study, an ABA-type BCP composed of poly(naphthalene diimide-bithiophene) (PNDI2T) and polyisobutylene (PIB) (PIB-*b*-PNDI2T-*b*-PIB) was shown to withstand up to 60% strain without cracking while still maintaining stable charge transport after stretching.^38^ These findings underscore the promise of BCPs as materials for flexible electronics, as they balance mechanical flexibility and electrical performance. Recently, Sato et al. proposed the first N-type multiblock conjugated BCPs comprising PDMS and PNDI2T; however, the BCP’s mobility significantly degraded after incorporating PDMS. In addition, there is no report about the mobility variations of N-type multiblock conjugated BCPs under strain.^39^ Therefore, BCPs can become promising materials for flexible electronics with judicious structural optimization.

In addition to the stretchability of polymer semiconductors, the flexibility of the electrodes is crucial to the overall performance of flexible electronic devices. Electrode materials must withstand mechanical stress without delaminating or peeling from the polymer semiconductors during deformation. While highly conductive, traditional metal electrodes are often brittle and prone to failure under mechanical stress. This challenge has led to innovative solutions for flexible and stretchable electrodes. For example, Lee et al. conducted resistance measurements using different thermal deposition rates and found that gold deposited at 0.1 Å s^–1^ exhibited the least resistance variation under strain and maintained good stability during cyclic testing. By integrating microcracked gold with stress relief layers (SRLs), they developed skin-like healthcare patches (SHPs) for biological sensing applications.^40^ Even after substantial mechanical stretching, the electrodes maintain minimal resistance, showcasing the significant potential of this technique for the advancement of flexible electronic devices. Remarkably, even after mechanical stretching, the resistance of these electrodes remains minimal. This technique, commonly referred to as “microcracked gold”, leverages the ductility of gold, which allows the electrode to maintain electrical conductivity despite the formation of microcracks during stretching.^41−43^ The densely packed gold nanoparticles on the surface and interior of the polymer matrix prevent the electrode from losing functionality even under extreme deformation. The development of microcracked gold electrodes represents a significant breakthrough in flexible electronics. By challenging traditional assumptions about metal electrodes, this innovation opens up new possibilities for their use in stretchable electronic devices. The ability of these electrodes to maintain their performance under large deformations enhances the stretchability and overall performance of flexible electronics, offering a promising direction for future research.

This study focuses on synthesizing and applying self-stretchable polymer semiconductors in OFETs. The polymer semiconductors are newly designed multiblock copolymers (multi-BCPs) composed of PNDI2T and PIB segments. Compared to previously reported ABA-type BCPs, where incorporating soft segments between rigid chains improves stretchability, the enhancement in stretchability was not very significant. Therefore, multi-BCPs were designed with a balanced ratio of soft and rigid segments in this study. The conjugated chains were embedded within the soft segments to minimize their impact on charge transport. For electrode fabrication, a microcracked gold electrode was developed by using low-rate thermal deposition to form infiltrated gold nanoparticles on the rubbery channel surface. Therefore, an integrated and intrinsically stretchable OFET device was fabricated. This integrated device can be used in biomedical electronics without toxic or corrosive electrode materials, such as SWCNTs or PEDOT:PSS. A key goal is to address the challenges of delamination and peeling of gold electrodes from the polymer channel during mechanical deformation. After stretching, the adhesion between the microcracked gold electrode and the multi-BCPs is explored to examine the relationship between electrode resistance, contact resistance, and channel mobility. Furthermore, the polymers’ optical properties and crystallographic parameters were investigated. These measurement methods were used to analyze the relationship between the physical and electrical properties of the polymer semiconductors, exploring the potential for integrating microcracked gold electrodes with polymer semiconductors.

## Experimental Section

### Materials

5-*tert*-Butyl-1,3-bis(1-chloro-1-methylethyl)benzene was synthesized according to the procedure described elsewhere.^44^ 5,5′-Bis(trimethylstannyl)-2,2’-bithiophene (2T, Sigma–Aldrich Japan K.K.) was purified by recrystallization using methanol. Thiophene was purified by distillation and stored in a refrigerator before use. 4,9-Dibromo-2,7-bis(2-decyltetradecyl)benzo[lmn][3,8]phenanthroline-1,3,6,8(2*H*,7*H*)-tetraone (Br-NDI-Br) was synthesized according to previous reports.^45^ The reference NDI-based polymer without PIB segments, poly{[*N*,*N*’-bis(2-decyltetradecyl)-naphthalene-1,4,5,8-bis(dicarboximide)-2,6-diyl]-*alt*-5,5′-(2,2’-dithiophene)} (PNDI2T) (*M*_n_ = 27,000, *M*_w_/*M*_n_ = 3.16), was synthesized according to the procedure described elsewhere.^38^ All other reagents and solvents were purchased from Sigma–Aldrich Japan K.K., Tokyo Chemical Industry Co., Ltd., Kanto Chemical Co., Inc., or FUJIFILM Wako Pure Chemical Industries, Ltd. These chemicals were used as received without further purification unless otherwise stated. The polymer solution used chloroform (99.9%) as a solvent purchased from Thermo Scientific. Trichloro(octadecyl)silane (OTDS) (>90%) and trichloroethylene (>99.5%) were purchased from Sigma-Aldrich. The PDMS substrate was a Sylgard 184 silicone elastomer agent from Dow Chemical.

### Characterizations

^1^H nuclear magnetic resonance (NMR) was documented using a JEOL JNM-ECX400 spectrometer at nuclear resonant frequencies of 400 or 600 MHz in chloroform-*d* (CDCl_3_) at 25 °C (for monomer precursors and monomers) or in 1,1,2,2-tetrachloroethane-*d*_2_ (C_2_D_2_Cl_4_-*d*_2_) at 100 °C (for polymers). The number-average molecular weight, weight-average molecular weight, and molecular weight distribution (*M*_n_, *M*_w_, and *M*_w_/*M*_n_) values of PIB derivatives were measured by size exclusion chromatography (SEC) using a JASCO GULLIVER HPLC system equipped with a pump (JASCO PU-4580), a column oven (JASCO CO-2065 Plus), and an RI detector (RI-1530). The column set was as follows: a guard column (Shodex K-G 4A) and two consecutive columns (Shodex K-804L, Shodex K-805L) eluted with tetrahydrofuran at 40 °C at a flow rate of 1.0 mL/min. The *M*_n_, *M*_w_, and *M*_w_/*M*_n_ values of multi-BCPs were measured by SEC using a JASCO GULLIVER HPLC system equipped with a pump (JASCO PU-4580), a column oven (JASCO CO–CO-1565), and a UV detector (UV, λ = 254 nm, JASCO UV-4575). The column set was as follows: a guard column (Shodex K-G 4A) and two consecutive columns (Shodex K-804L, Shodex K-805L) eluted with chloroform at 40 °C at a flow rate of 1.0 mL/min. Polystyrene standards were employed to prepare calibrations for all SEC experiments. Thermal gravimetric analysis (TGA) was performed using TGA 4000 (PerkinElmer) or TG/DTA6200 equipped with EXSTAR 6000 (Hitachi High-Tech) with a heating rate of 10 °C min^–1^ under a nitrogen flow. Differential scanning calorimetry (DSC) analysis was conducted using a DSC6200 equipped with an EXSTAR 6000 (Hitachi High-Tech) with a ramping rate of 10 °C min^–1^ under a nitrogen flow. The UV–vis absorption spectra of the polymer films were obtained using a V-770 UV–visible/NIR spectrophotometer (JASCO, Inc.) with an operational wavelength range of 200–1200 nm. Cyclic voltammetry (CV) was conducted with a CHI 6273E electrochemical analyzer in a three-electrode cell system, where the working electrode was an ITO glass spin-coated with a polymer film, a platinum foil served as the counter electrode, and an Ag/AgCl electrode acted as the reference electrode in a nonaqueous electrolyte system comprising 0.1 M tetrabutylammonium perchlorate in acetonitrile. The polymer film morphology and force mapping were examined with an AFM100plus (Hitachi), with surface morphology in tapping mode and mapping force in scanning intelligent scan (SIS) model using Derjaguin–Muller–Toporov (DMT) full model fitting. The thickness of the polymer film was measured using the Alpha-step D-300 (KLA-Tencor/AS-IQ). Grazing-incidence X-ray diffraction (GIXD) analysis was conducted at the TLS BL23A1 beamline of the National Synchrotron Radiation Research Center (NSRRC) in Taiwan, with polymer films spin-coated on silicon wafers, using an incidence angle of 0.2° and a monochromatic X-ray wavelength of 1.24 Å. The GIXD parameter calculation method follows: lamellar stacking distance (*d*_100_) = 2*π/q*, coherence length (*L*_c_) = 0.9 × 2*π/*FWHM, paracrystalline disorder , and the relative degree of crystallinity (rDoC) of polymer films under tensile strains is derived from the integral area of (100) diffraction in comparison to that at 0% strain. The OFET device properties were evaluated in a nitrogen-purged glovebox using a Keithley 4200-SCS semiconductor parameter analyzer (Keithley Instruments, Inc.). The charge mobility (μ_e_) and threshold voltage (*V*_th_) were calculated following the slope or extrapolation of the square root of drain current (*I*_d_^1/2^) versus gate voltage (*V*_g_) in the saturation region of the transfer curve: . The transfer curve parameters were set at *V*_d_ = 100 V and *V*_g_ scanned from −20 to 100 V, while the output curve parameters were set with *V*_d_ = −20 to 100 V and *V*_g_ scanned from 0 to 100 V. The contact resistance measurement is detailed in the Supporting Information.

### Synthesis of Multi-BCPs

A typical synthesis of mAB73 is described in detail below. A two-necked 50 mL round-bottom flask was charged with 2T (0.0482 g, 0.098 mmol) and Br-NDI-Br (0.101 g, 0.092 mmol). After dissolving these compounds in deoxidized toluene (5 mL), N_2_ was bubbled through the solution for 30 min. A deoxidized toluene solution (5 mL) of tris(dibenzylideneacetone)dipalladium(0) (Pd_2_(dba)_3_, 0.0088 g, 0.0096 mmol) and tri(*o*-tolyl)phosphine (P(*o*-tol)_3_, 0.0278 g, 0.0913 mmol) was then added to the mixture. The solution was stirred under reflux conditions for 1 h. In a separate two-necked flask, α,ω-chain-end-functionalized PIB with 5-bromothien-2-yl groups (BrT-PIB-TBr, 0.0455 g, 0.0063 mmol, *M*_n_ (SEC) = 7,200, Scheme S2, Figure S1) was dissolved in 10 mL of deoxygenated toluene and bubbled with N_2_ for 30 min. The BrT-PIB-TBr solution was added to the polymer solution and then stirred under reflux conditions for 24 h. The end-capping reaction was performed by adding 2-bromothiophene (4 μL) and 2-(tributylstannyl)thiophene (7 μL), respectively, and stirring under reflux conditions for 2 h for each. After the reaction, a small amount of sodium dimethyldithiocarbamate dihydrate was added to the solution. The resulting solution was poured into methanol to precipitate the polymer, filtered off, purified by Soxhlet extraction with methanol, acetone, and hexane, and recovered with chloroform (each for 24 h). After chloroform was removed under reduced pressure, the polymer was freeze-dried from its benzene solution to afford mAB73 as a dark blue polymer. Yield = 66 mg (45%), *M*_n_ (SEC) = 35,500, *M*_w_/*M*_n_ (SEC) = 2.20.

^1^H NMR (600 MHz, C_2_D_2_Cl_4_-*d*_2_, 100 °C, Figure S6): δ (ppm) 8.87 (s, 2H), 7.40 (s, 4H), 4.17 (s, 4H), 2.08 (s, 2H), 1.53–1.47 (m, 29H), 1.42–1.28 (m, 95H), 1.17 (s, 43H), 0.90 (s, 15H).

mAB60: A similar synthetic protocol was employed as for mAB73, except for using different comonomer ratios, 2T (0.0529 g, 0.108 mmol), Br-NDI-Br (0.102 g, 0.093 mmol), and BrT-PIB-TBr (0.111 g, 0.0154 mmol). Yield = 140 mg (67%), *M*_n_ (SEC) = 42,800, *M*_w_/*M*_n_ (SEC) = 2.10.

^1^H NMR (600 MHz, C_2_D_2_Cl_4_-*d*_2_, 100 °C, Figure S7): δ (ppm): 8.86 (s, 1H), 7.38 (s, 3H), 4.03–4.26 (4H), 2.01–2.10 (2H), 1.51–1.45 (m, 54H), 1.33 (d, 80H), 1.17–1.11 (m, 80H), 0.87 (d, 13H).

### Fabrication of the OFET Devices

The OFET device was assembled in a bottom-gate/top-contact configuration, with a conjugated polymer film as the charge transport channel and a 300 nm-thick SiO_2_ layer as the dielectric (areal capacitance = 10.5 nF cm^–2^). The 5 mg mL^–1^ polymer solution in chloroform, used as the solvent, was spin-coated onto the ODTS-modified silicon wafer. The polymer films underwent thermal annealing at 200 °C for 30 min in a nitrogen-filled glovebox and were then gradually cooled to room temperature. Subsequently, a 40 nm-thick gold layer was thermally deposited at 0.5 Å s^–1^ onto the polymer film through a shadow mask to define the drain/source contact electrodes, forming a channel with a length (*L*) of 50 μm and a width (*W*) of 1000 μm.

The film-transfer technique was employed to investigate the polymer’s stretchability and mobility characteristics. Spin-coated polymer films on the OTDS-modified silicon wafer were peeled off using a PDMS slab with a base/cross-linker = 20/1 w/w. The PDMS substrate was stretched to the desired strain ratio and transferred onto a 300 nm-thick SiO_2_ wafer. A 40 nm-thick gold electrode was thermally deposited onto the polymer, with a mask applied to cover the polymer.

### Fabrication of the Integrated Stretchable Devices

The fabrication process of the integrated stretchable device differs from that of the OFET device in terms of the order and conditions of thermal deposition. First, a polymer film was spin-coated on a 300 nm SiO_2_ wafer modified with OTDS and masked before undergoing low-rate thermal deposition (0.1 Å s^–1^) to create 40 nm-thick gold electrodes. The polymer and gold electrodes were peeled off together by using a PDMS slab. The PDMS/gold/polymer channel stacks, arranged from top to bottom, were stretched to the desired strain ratio and transferred onto a 300 nm-thick SiO_2_ wafer.

## Results and Discussion

### Synthesis of Multi-BCPs

**Scheme 1:**
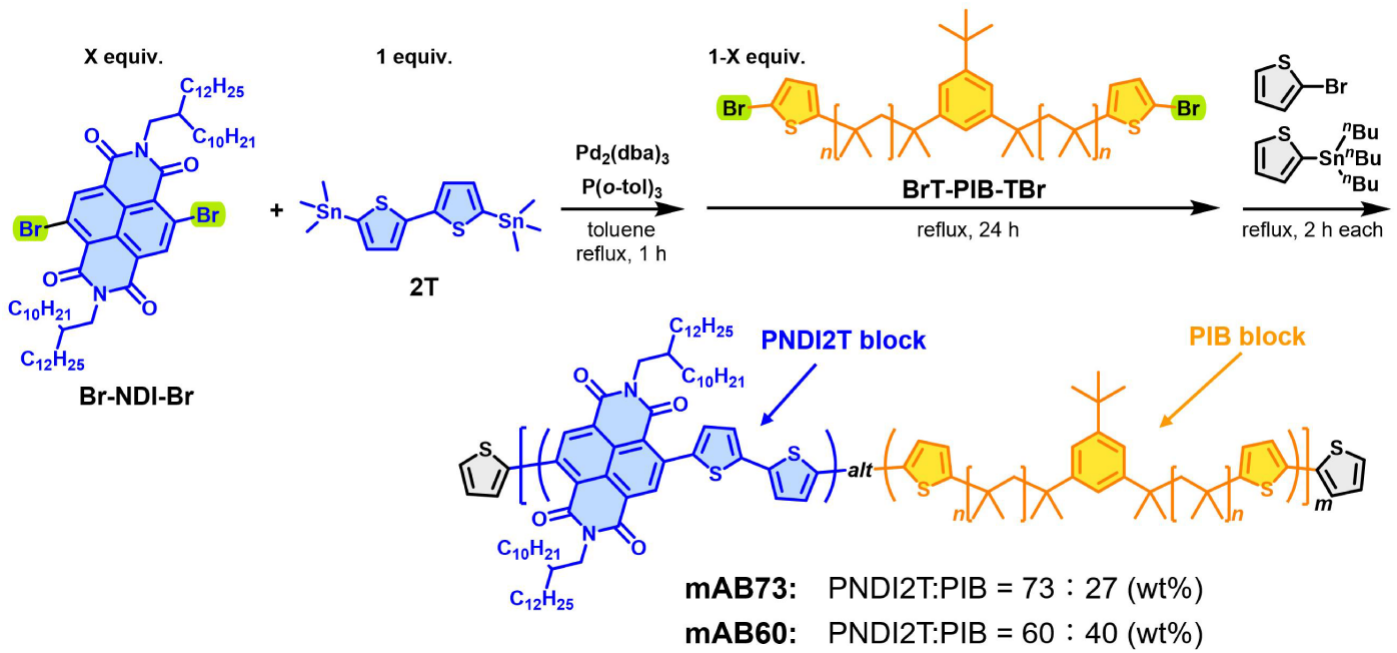
Synthetic Routes for the Conjugated Multiblock Copolymers: mAB73 and mAB60

We previously synthesized PIB-*b*-PNDI2T-*b*-PIB by the Migita-Kosugi-Stille coupling polycondensation of Br-NDI-Br and 2T, followed by a polymer coupling reaction with ω-chain-end-functionalized PIB with a 5-bromothien-2-yl group (PIB-TBr).^38^ In this study, a novel α,ω-chain-end-functionalized PIB with 5-bromothien-2-yl groups, BrT-PIB-TBr, was synthesized by bromination of α,ω-chain-end-functionalized PIB with thien-2-yl groups (T-PIB-T) with *N*-bromosuccinimide and used in a similar polymer coupling reaction instead of PIB-TBr (Schemes S1 and S2). BrT-PIB-TBr possessed an *M*_n_ value of 7,200 and an *M*_w_/*M*_n_ value of 1.11, as confirmed by SEC (Figure S1). Note that the narrow distributions almost remain unchanged after the bromination reaction. In addition, the quantitative conversion from the thien-2-yl to the 5-bromothien-2-yl group is confirmed by ^1^H NMR (Figures S2 and S3). The synthesis of multi-BCPs composed of PNDI2T and PIB segments, mAB73 (PIB: 27 wt %) and mAB60 (PIB: 40 wt %), was performed by Migita-Kosugi-Stille coupling polycondensation of Br-NDI-Br and 2T in the presence of Pd_2_(dba)_3_ and P(*o*-tol)_3_ in toluene at reflux temperature for 1 h, followed by in situ reaction with varied molar ratios of BrT-PIB-TBr (toward Br-NDI-Br) for 24 h (Scheme 1). The synthetic results are summarized in Table 1. The *M*_n_^SEC^ values for mAB73 and mAB60 were determined to be 35,500 and 42,800, respectively, as determined by SEC (Figures S4 and S5). Indeed, a clear shift in the top peak to a higher molecular weight region is observed in the SEC UV traces for multi-BCPs compared to those for the precursor first block of PNDT2T (*M*_n_^SEC^ = 16,000 for mAB73 and *M*_n_^SEC^ = 10,000 for mAB60) and BrT-PIB-TBr (*M*_n_^SEC^ = 7,200), indicating high blocking efficiency. The weight compositions of the PNDI2T and PIB segments were determined by ^1^H NMR to be 73:27 (mAB73) and 60:40 (mAB60) based on the intensity ratio between specific signals of four *N*-methylene protons ***a*** and those of six methyl protons ***g*** assigned to the monomer repeating units of PNDI2T and PIB segments, respectively (Figures S6 and S7).

**Table 1:** Synthetic Results of the Studied Polymers, Including Their Molecular Weight and Composition Parameters, Derived from the SEC and NMR Analyses

	PNDT2T		BrT-PIB-TBr		Multiblock copolymer		
Polymer	*M*_n_^a^	*M*_w_/*M*_n_^a^	*M*_n_^a^	*M*_w_/*M*_n_^a^^a^	*M*_n_^a^	*M*_w_/*M*_n_^a^^a^	Composition (PNDI2T:PIB)^b^
**NDI**	27,000	3.14	N/A	N/A	N/A	N/A	100:0
**mAB73**	16,000	1.82	7,200	1.11	35,500	2.20	43:27
**mAB60**	10,000	1.29	7,200	1.11	42,800	2.10	60:40

a Determined by SEC in chloroform at 40 °C, based on a calibration using polystyrene standards.

b Determined by ^1^H NMR in C_2_D_2_Cl_4_ at 100 °C, based on the signal intensity ratio for each block segment.

### Thermal and Optical Properties of Multi-BCPs

The thermal properties of the studied polymers were characterized by TGA (Figure S8) and DSC (Figures S9 and S10). Despite the incorporation of aliphatic PIB segments in the main chains, multi-BCPs show high thermal stability, with 5 wt % weight loss temperatures (*T*_d_^5%^) of over 400 °C (mAB73: *T*_d_^5%^ = 402 °C; mAB60: *T*_d_^5%^ = 401 °C), probably due to the high heat resistance of the PIB elastomer, which consists of saturated hydrocarbons, similar to PIB-*b*-PNDI2T-*b*-PIB previously reported.^37^ mAB73 showed an exothermic peak at 46 °C in the DSC thermogram, probably assignable to the glass transition temperature (*T*_g_) of PNDI2T. The typical *T*_g_ of PNDI2T is around 100 °C.^48^ It is speculated that PIB in the BCP structure makes PNDI2T more flexible. The literature indicates the heating profile after cooling from 70 °C annealing. However, in this study, bulk samples were measured directly without thermal pretreatment. Therefore, the interference of PIB and the absence of annealing-induced crystallization led to a decrease in *T*_g_ of PNDI2T in mAB73. mAB73 showed an exothermic peak at 258 °C in the DSC thermogram, probably assignable to the melting temperature (*T*_m_) of phase-separated crystalline PNDI2T domains. In contrast, the exothermic peak almost disappeared for mAB60, probably due to the lower PNDI2T composition and the lower crystallinity of PNDI2T domains. Instead, a new transition peak around −10 °C was observed, probably *T*_g_ (or *T*_m_) of phase-separated PIB domains with a higher composition than mAB73. Figure S11a–d shows CV results of the multi-BCP thin films for calculating their highest occupied/lowest unoccupied molecular orbitals (HOMO/LUMO) levels. The UV–Vis absorption spectra of the multi-BCP thin films are presented in Figure S11e, and the optical parameters are summarized in Table S1. The mAB60 film showed slightly smaller charge-transfer absorption than the mAB73 film, probably due to the lower intermolecular interaction of PNDI2T segments by increasing PIB composition. Nevertheless, both films present almost similar optical properties, indicating that PIB’s incorporation has little effect on the energy bandgap of multi-BCP in the thin-film state (Figures S11f).

### Morphological Analysis of the Multi-BCP Thin Films

**Figure 1:**
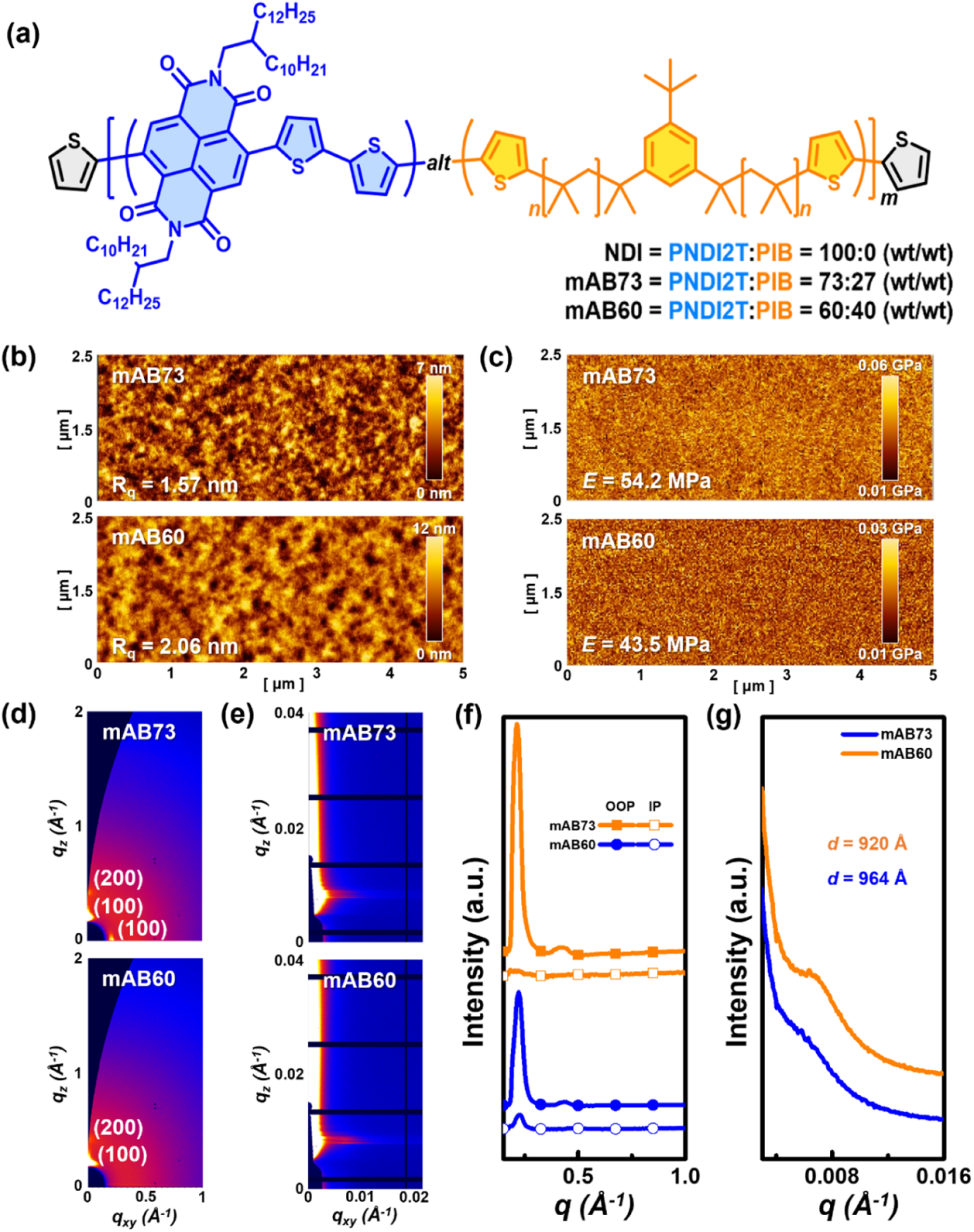
(a) Chemical structure of the multiblock copolymers studied. (b) AFM topographies, (c) mechanical mappings, (d) 2D GIWAXS, (e) GISAXS patterns, (f) 1D GIWAXS profiles in the OOP (solid symbols) and IP (open symbols) directions, and (g) 1D GISAXS profiles integrating along the IP direction of the regular polymer films on wafer substrates.

The N-type multi-BCPs in this study comprise alternating rigid PNDI2T and flexible PIB blocks. The chemical structures of the multi-BCPs are shown in Figure 1a, and the polymers are named based on their weight percentages: NDI (0 wt %), mAB73 (27 wt %), and mAB60 (40 wt %). These polymers’ surface morphology and mechanical mappings were analyzed using AFM, as shown in Figure 1b, c. The roughness and modulus values for mAB73 and mAB60 are 1.57 nm, 54.2 MPa, and 2.06 nm, 43.5 MPa, respectively. The morphology of mAB73 is smoother than that of mAB60 due to the lower content of flexible chain segments, which reduces phase separation during film formation. In contrast, mAB60, with a higher proportion of flexible chains, exhibits a smaller modulus and more pronounced surface roughness. Incorporating flexible chains significantly alters the polymers’ surface morphology and mechanical properties.

AFM was primarily used to investigate the surface morphology of the films, while GIWAXS and GISAXS were employed to examine the crystallinity and domain size of the polymers. Figure 1d,e shows the 2D patterns of GIWAXS and GISAXS, and Figure 1f,g presents the corresponding 1D line-cutting profiles. The GIWAXS data reveal that both polymers exhibit lamellar stacking with (n00) signals aligned along the out-of-plane (OOP) direction and weak diffraction along the in-plane (IP) direction. The values of the OOP *d*_100_ and *L*_c_ for mAB60 are 29.2 and 133.6 Å, respectively, higher than those of 28.3 and 118.2 Å for mAB73. Moreover, the paracrystalline disorder (*g*) of mAB60 (0.177) is lower than that of mAB73 (0.185), indicating that introducing more flexible segments into mAB60 leads to an ordered arrangement among the conjugated segments, resulting from phase separation. In the 2D GISAXS pattern, the IP integration yields the 1D profile, where a domain peak is observed. The domain distances (*d*) for mAB73 and mAB60 are 964 and 920 Å, respectively. The domain peak of mAB60 is sharper and more substantial than that of mAB73. This suggests that the higher flexible chain content in mAB60 promotes phase separation, leading to a more robust microphase separation. Therefore, introducing flexible chain segments significantly impacts the surface morphology, mechanical properties, self-assembly, and crystallographic parameters of the multi-BCPs.

### Morphological Analysis of the Stretched Polymer Films

**Figure 2:**
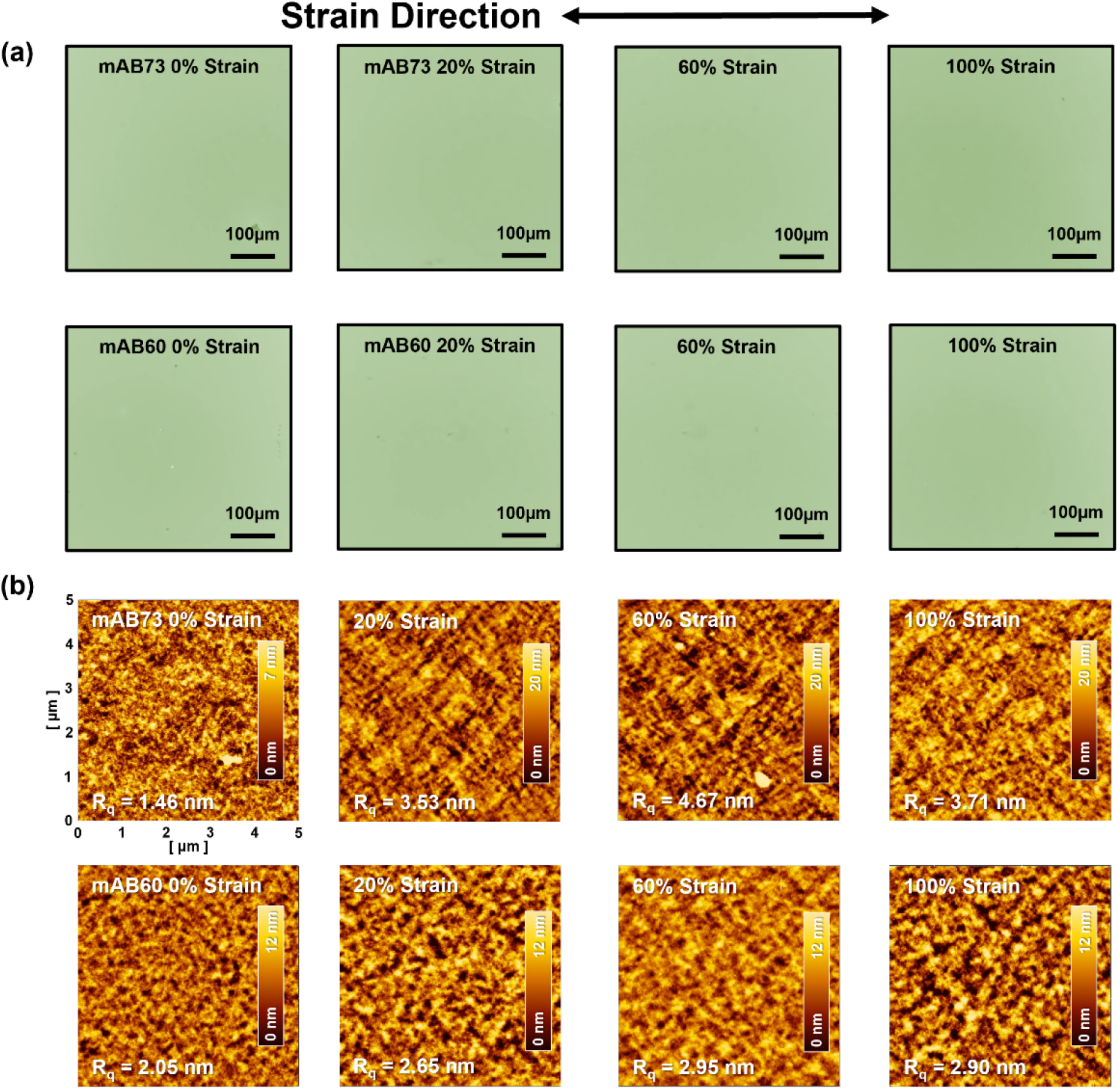
(a) OM images and (b) AFM topographies of the stretched polymer films. Note that the OM images represent the polymer morphology on a stretched PDMS slab, and the AFM morphologies indicate those transferred back to the silicon wafer.

After analyzing the regular polymer thin film without tensile strain, the study further investigates the surface morphology and crystallinity of the polymer after stretching. Figure 2a shows the OM images of polymer films on a PDMS substrate under different strains. It was observed that when the polymer film was stretched to 100%, no cracks appeared. Figure 2b shows the surface morphology of AFM height images. The roughness of mAB73 is higher than that of mAB60. Both polymer films remained consistent after stretching, demonstrating that the multi-BCPs possess good elasticity.

**Figure 3:**
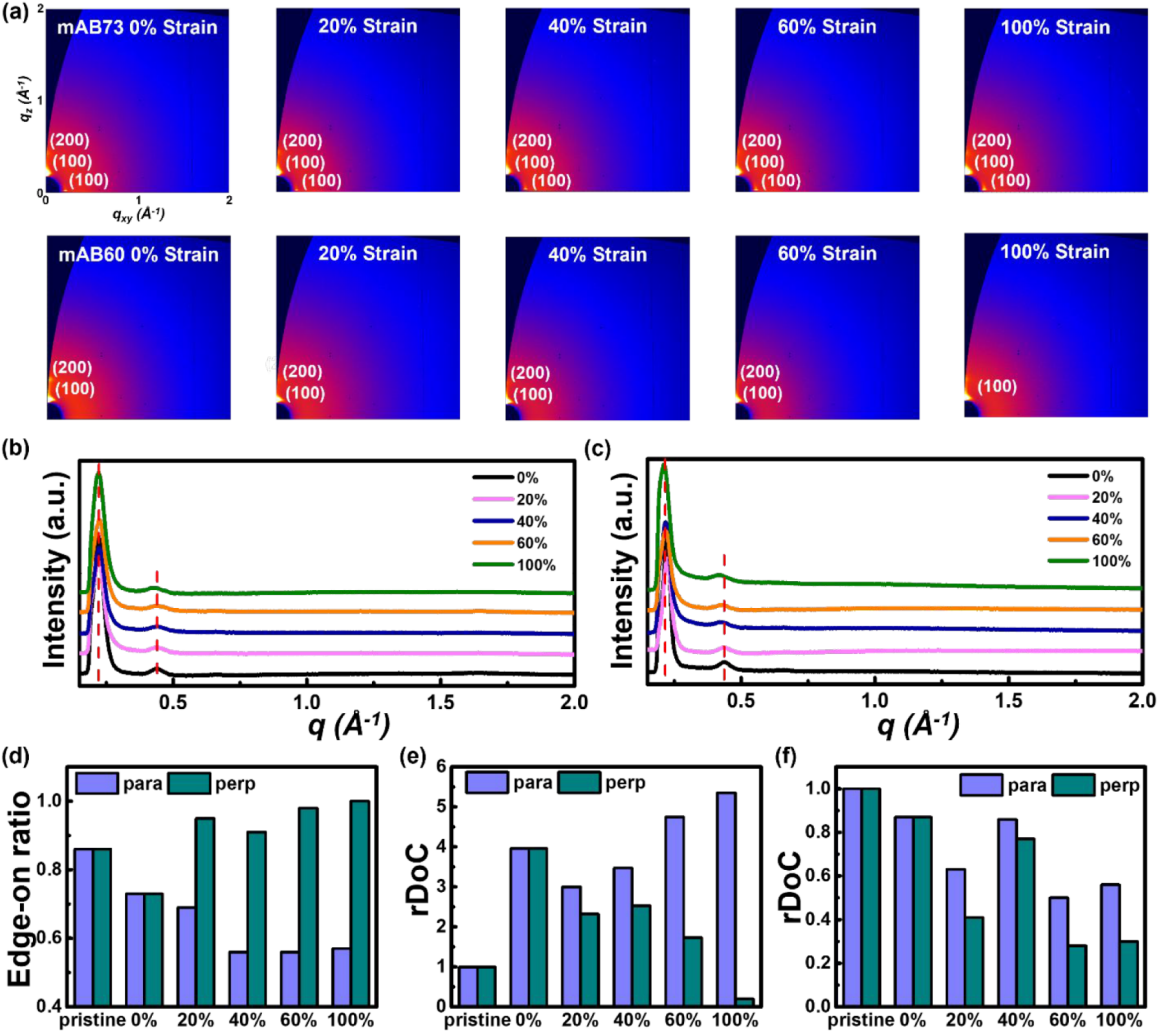
(a) 2D GIWAXS patterns of the transferred/stretched mAB73 (top) and mAB60 (bottom) polymer films at different strain ratios, with the stretching direction parallel to the incident beam. 1D GIWAXS profile for the OOP direction of the transferred/stretched (b) mAB73 and (c) mAB60 films at different strain ratios, with the stretching direction parallel to the incident beam. (d) The edge-on ratio of the mAB73 films at varied strain levels. Note that mAB60 shows no signal in the IP direction; therefore, under different stretching ratios, it becomes edge-on. The relative degree of crystallinity (rDoC) for (e) mAB73 and (f) mAB60 films at varied strain levels.

The crystallinity of the polymer thin film was further explored using GIWAXS and GISAXS. The 2D GIWAXS patterns show that the polymer’s orientation remained almost unchanged after stretching at different strain ratios parallel (Figure 3a) or perpendicular (Figure S12) to the incident X-ray beam. Figures 2b, 2c, and S13 show the 1D GIWAXS profiles for the multi-BCP films integrated along the OOP and IP directions, with the stretching direction aligned parallel and perpendicular to the incident beam. The crystallographic parameters are summarized in Tables S2 and S3. When stretched in the direction parallel to the X-ray beam, the OOP (200) peak of mAB73 and mAB60 shifts to the left under 100% strain, indicating that both polymers undergo strain-induced developments to the crystallographic parameters, and mAB60 showing more pronounced variations. Further analysis of the changes in crystallinity parameters reveals that, under parallel strain, the OOP direction of mAB73 showed that *d*_100_ compressed and then expanded, and *g* increased and then decreased, indicating structural disorders at low strains and recovery at 60–100% strains. For mAB60, *d*_100_ and *g* increased monotonically with strain levels of 0–100%, showing that the higher number of soft segments makes the multi-BCP vulnerable to strains, causing continuous changes in crystallographic parameters.

In the perpendicular direction, mAB73 showed no clear trend, but its structural order gradually decreased. Similarly, mAB60 did not exhibit a clear trend in the perpendicular case, though *g* increased, and its crystallinity arrangement was similar to that of the parallel case. In the IP direction, mAB73 shows signals in the parallel direction similar to the OOP direction, with significant structural changes occurring under 60–100% strain, indicating an elastic limit in stress accommodation. In the perpendicular direction, the signal gradually decreases with stretching and disappears at 100%, suggesting a transition from a face-on to edge-on orientation during stretching, as shown in Figure 3d. In contrast, mAB60 exhibits no signals in the IP direction, indicating a predominant edge-on orientation. Next, Figure S14 shows the pole figure for the (100) diffraction peaks of multi-BCP films at different strain levels, with the stretching direction aligned both parallel and perpendicular to the incident beam. The rDoC of the multi-BCPs is summarized in Figure 3e,f. The results show that for mAB73 in the parallel case, rDoC increases with increasing strain, while mAB60 decreases. Therefore, considering the crystallographic parameter variations, mAB73 is more resilient to tensile stress than mAB60.

**Figure 4:**
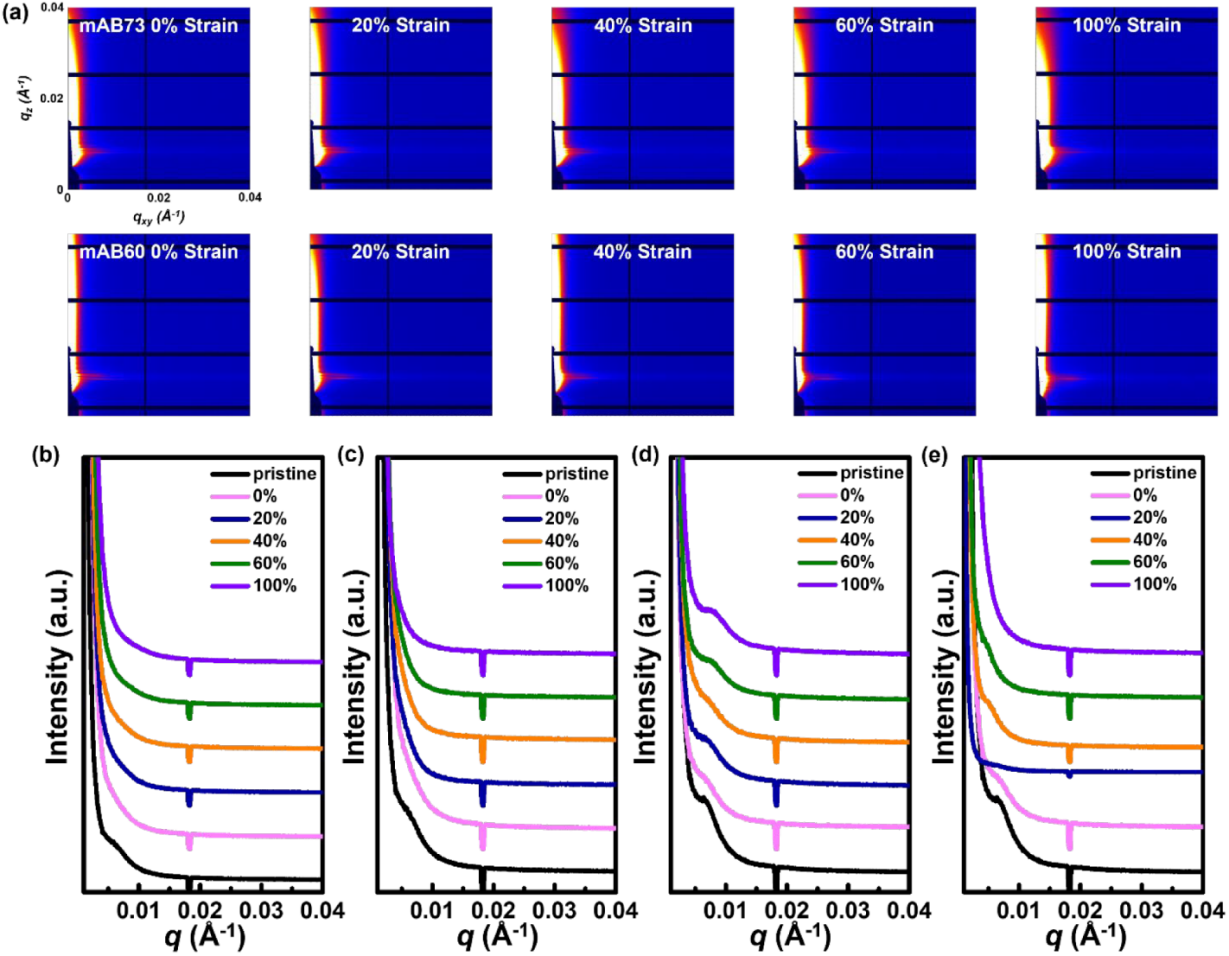
(a) 2D GISAXS patterns of the transferred/stretched mAB73 (top) and mAB60 (bottom) films at different strain ratios, with the stretching direction parallel to the incident beam. 1D GISAXS line-cutting profiles of (b, c) mAB73 and (d, e) mAB60 polymer films at different strain ratios with the stretching direction (b, d) parallel and (c, e) perpendicular to the incident beam.

The block copolymer architecture generates microphase separation, which is also influenced by tensile strains. Figures 4a and S15 show the 2D GISAXS patterns for the BCP films at different strain ratios, with the stretching direction aligned parallel and perpendicular to the incident beam. The 1D line-cutting profiles are shown in Figure 4b–e, and the domain distances (*d*) are summarized in Table S4. It was observed that mAB73 exhibited signals only in the regular film, whereas mAB60 showed domain peaks at 100% stretching. The higher content of soft segments tends to form a self-assembly with conjugated and soft segments, leading to phase separation. As the strains increased, *d* also increased, as stress caused the aggregated segments to be pulled apart while the soft segments endured the stress. Even at 100% stretching, domain peaks still existed. Additionally, compared to the AFM roughness, the high aggregation of mAB60 makes the chain segments less susceptible to being stretched, resulting in a minor increase in roughness. In contrast, during the stretching process of mAB73, the chain segments are more easily elongated, leading to higher roughness than mAB60. In summary, introducing soft segments effectively enhanced the stretchability of the polymer, allowing it to withstand more stress and reducing changes in the crystalline regions. However, introducing excessive soft segments leads to phase separation in the polymer thin film, potentially deteriorating the charge transport stability under strain.

### Device Characteristics of the Stretched Polymer Films

**Figure 5:**
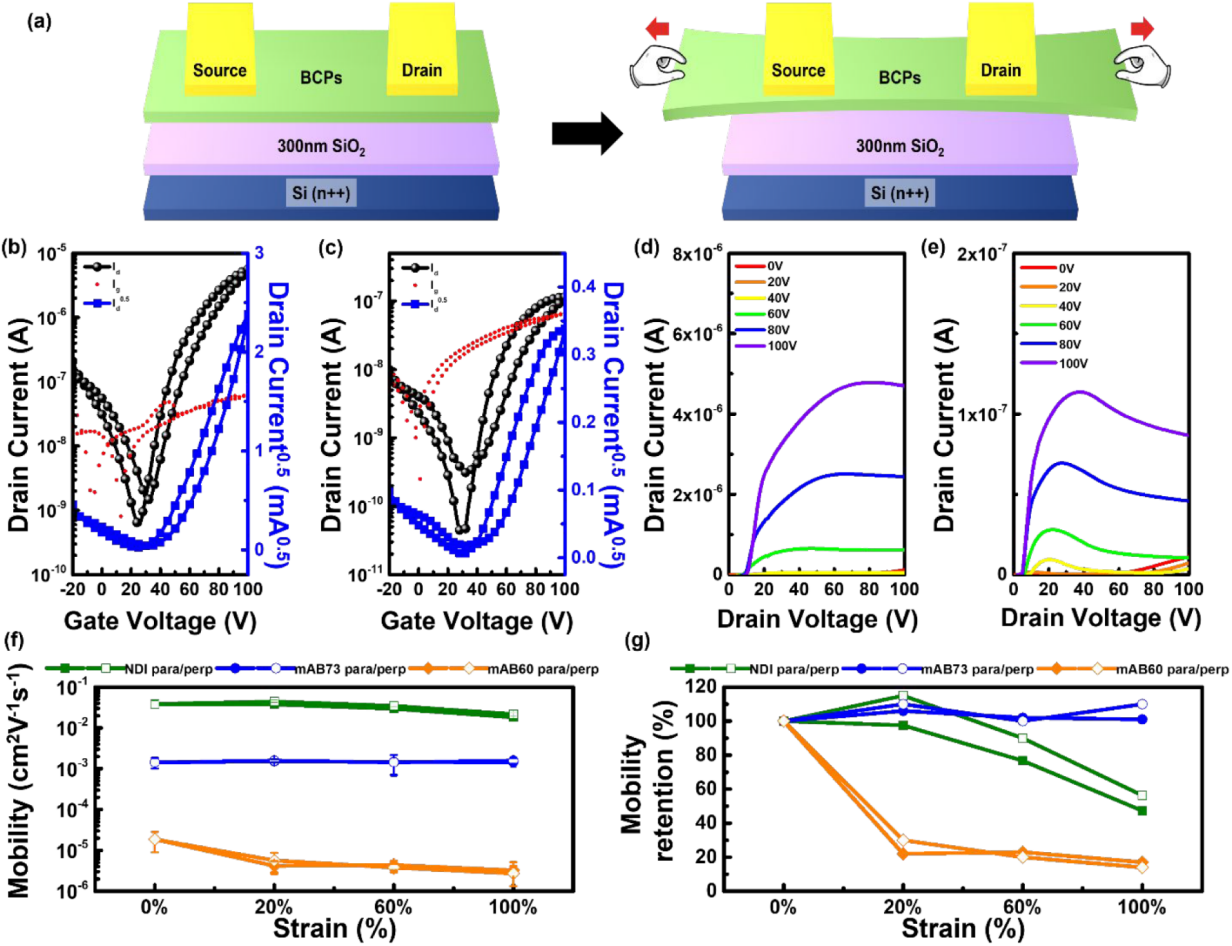
(a) Schematic diagram of OFETs with stretched/transferred polymer semiconductor onto a 300 nm SiO_2_ wafer. OFETs (b, c) transfer and (d, e) output curve for the regular (b, d) mAB73 and (c, e) mAB60 films. Correlation of (f) mobility and (g) mobility retention to different tensile strain ratios applied to the polymer films. Note that “para” or “perp” indicates that the strain is parallel or perpendicular to the channel direction.

The multi-BCPs are applied to OFET devices with a bottom-gate/top-contact configuration, as shown in Figure 5a, to investigate the electrical performance of the polymer films after stretching. Figures 5b–d and S16 present the transfer and output curves of the OFETs with the regular polymer films. The OFET device parameters of regular polymer films are listed in Table S5. The charge mobilities for NDI, mAB73, and mAB60 in the regular OFETs are 0.091, 0.017, and 5.6 × 10^–4^ cm^2^ V^–1^ s^–1^, respectively. As the number of soft segments increases, mobility gradually decreases. From the output curve, mAB73 shows a stable current in the saturation region, while mAB60, which contains more soft segments, does not exhibit a stable current and shows a decline, indicating its bias and thermal instability.

The film-transfer technique was applied to fabricate the OFET devices to investigate the mobility–stretchability relationship of multi-BCPs. Figure S17 shows the transfer curves of the BCP films at different strain levels, and their OFET parameters are summarized in Table S6. Figure 3f,g shows the mobility and retention at different strain ratios. The mobility values for NDI, mAB73, and mAB60 at 0% strain are 0.039, 0.0014, and 1.9 × 10^–5^ cm^2^ V^–1^ s^–1^, respectively. As the strain increases, the charge mobility for NDI and mAB60 gradually decreases, while mAB73 maintains stable performance. Mobility retention was calculated based on the 0% strain values to observe the strain-mobility behavior better. At 100% strain, the mobility retention for NDI, mAB73, and mAB60 in the stretched direction parallel/perpendicular to the channel is (47, 56)%, (101, 110)%, and (17, 14)%, respectively. It was observed that mAB73 exhibited stable electrical performance during stretching. Exploring the relationship between rDoC and charge mobility, it can be seen that the rDoC of mAB73 increases in the parallel direction as the strain increases. The soft segments can withstand stress, ensuring that the conjugated segments in the crystalline regions remain unaffected, thus maintaining stable electrical performance after stretching. In contrast, the rDoC of mAB60 decreases with increasing strain, thereby deteriorating its electrical performance. In the strain-dependent charge carrier mobility measurements, mAB73 exhibits slightly higher mobility in the vertical direction than in the horizontal direction. Although rDoC decreases in the vertical direction as the strain increases, the crystalline orientation gradually transitions from a face-on to an edge-on arrangement under stress. This transition enhances charge carrier mobility in the perpendicular direction, effectively counteracting the reduction in mobility caused by a decrease in rDoC. Additionally, the aggregation between π–π stacks and soft segments specifically impacts charge transport. From GISAXS analysis, mAB60 exhibits a higher degree of aggregation, where the aggregation of soft segments disrupts charge transport between π–π stacks. This results in mAB60 having lower charge mobility than the other two polymers. From the crystallographic, aggregation, and electrical properties, it is confirmed that among the BCPs with different PIB ratios, mAB73 performs the best. It exhibits excellent ductility in mechanical properties and maintains good charge mobility under strain.

Compared to previously reported ABA-type BCPs,^38^ the interwoven soft and rigid segments in multi-BCPs exhibit superior performance in elastic modulus and crack-onset strain, indicating better mechanical properties. However, with regard to regular charge mobility, multi-BCPs perform worse than ABA-type BCPs due to hindered charge transport between rigid segments. Nevertheless, the interwoven soft and rigid segments in multi-BCPs effectively withstand mechanical stress, resulting in highly stable charge mobility under strain. In contrast, the ABA-type structure, where soft segments encapsulate rigid segments, exhibits a more rigid overall structure, leading to inferior charge mobility stability under strain compared to that of AB-type BCPs. Our experimental results show that multi-BCPs are promising materials for flexible electronics, demonstrating stable electrical performance under tensile stress and excellent tolerance to mechanical deformation.

### Device Characteristics of the Multi-BCP Thin Films with Microcracked Gold

**Figure 6:**
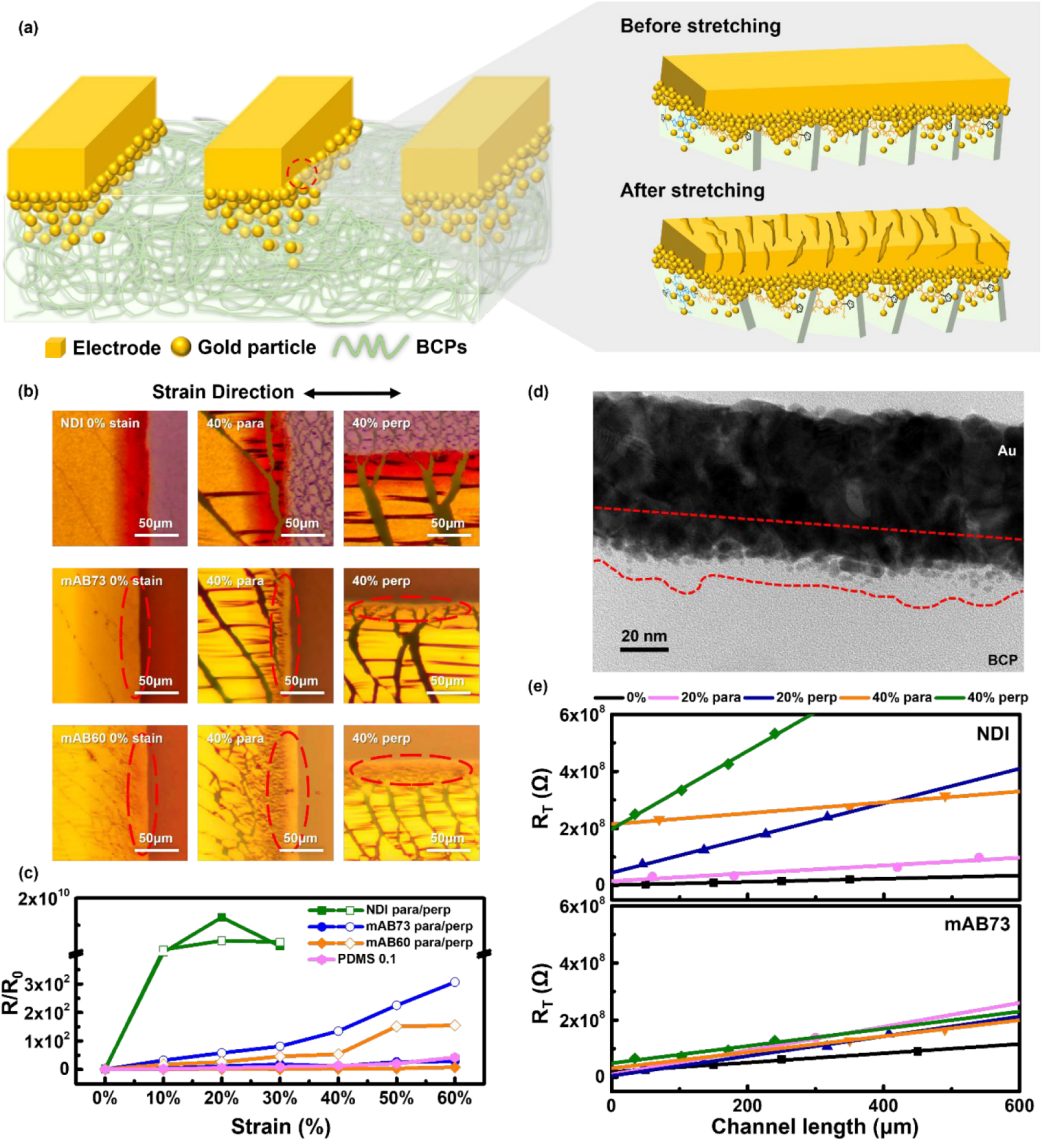
(a) Schematic diagram of polymer films with microcracked gold as contact electrodes for intrinsically stretchable OFET device. (b) OM images of polymer films with microcrack gold on PDMS. Note that the microcracked gold film occurs at the side of gold electrodes as labeled by the red circles. (c) The electrical resistance of gold electrodes on the polymer films with different strains. (d) Cross-sectional TEM images of Au electrodes deposited on BCP film at 0.1 Å s^–1^. (e) The total resistance at *V*_d_ = 20 V and *V*_g_ = 80 V of NDI (top) and mAB73 (bottom) with varying channel lengths for extracting the contact resistance.

Integrating polymer channels and contact electrodes warranting high stretchability is favorable for fulfilling stretchable electronic devices, and the microcracked gold technique provides a potential strategy to combine them.^46,47^ Therefore, the multi-BCPs were integrated with microcracked gold to form a stretchable semiconductor polymer and electrode system. First, we measured the resistance change after stretching by depositing gold electrodes on the stretchable soft substrate PDMS using different deposition rates, as shown in Figure S18. This verifies that microcracked gold formed under low deposition rates exhibits excellent stretchability. Next, gold electrodes were deposited on the polymer film by using a low-rate thermal deposition method. The films were transferred/stretched to the required strain and then reattached to the substrate for analysis. The performance of the OFET device in response to strain is investigated, as illustrated in Figure 6a. Morphological observations indicate the cracking behavior of the gold electrodes under strain on the polymer films, as shown by the OM images in Figures 6b, S19, and S20. Cracks are evident on electrodes deposited on NDI films, where the gold-polymer interface is relatively fragile, making the gold electrode susceptible to breakage upon stretching. Gold nanoparticles penetrate the more loosely structured multi-BCPs when soft segments are introduced into the conjugated polymer, enhancing the material’s ductility due to the rubbery support. Notably, no fractures are observed at the edges of the electrodes on mAB73 and mAB60 films with soft segment incorporation, preserving the channel continuity under strain.

Further analysis of strain-induced resistance changes Figure 6c shows that the resistance of mAB73 and mAB60 increases approximately 200–400 times with strain, while NDI’s resistance dramatically rises by several orders of magnitude. Notably, resistance increases more significantly under perpendicular strain than under parallel strain due to the tensile and compressive stress applied to the gold electrode. mAB60 contains more soft segments and exhibits lower resistance in both directions than mAB73. Additionally, the resistance variation of both BCPs is comparable to that of PDMS, indicating that the microcracked gold does not introduce additional resistance that could affect the performance of the BCPs. Figure 6d shows the focused ion beam-transmission electron microscopy (FIB-TEM) image of the interface between the microcracked gold and BCP. From the TEM images, gold infiltration into the BCP film was observed. Under low-rate thermal deposition, gold diffuses into the polymer layer. Additionally, introducing the soft PIB segment increases the structural looseness of the BCP, facilitating gold diffusion into the polymer. Gold particles tend to aggregate, forming Au clusters that enhance stability.^49,50^ This aggregation behavior is evident in the TEM images. The diffusion of gold into the BCP improves adhesion between the gold electrode and the polymer, ensuring a stable electrical performance during stretching.

Next, the channel and contact resistances are considered to understand the electronic properties of the integrated polymer channel and contact electrode system. Using the transfer line method (TLM), measure the transfer curves by constant *V*_d_ while varying the channel length and *V*_g_ to calculate the total resistance, which is the sum of the channel and contact resistances.^47^ Intercepts from total resistance measurements provide contact resistance and channel resistance values, as shown in Figures 6e and S21, with values compiled in Tables S7 and S8.The relevant calculation equations are listed in the Supporting Information. Experimental observations indicate that contact resistance converges at higher voltages (*V*_g_ = 50–80 V). Initially, NDI and mAB73 exhibit similar contact resistance at 0% strain. However, as the strain ratio increases, the contact resistance of NDI rises sharply, whereas that of mAB73 remains relatively stable. An interesting finding is that NDI exhibits a significant difference in resistance slope under strain between the parallel and perpendicular directions. The slope represents the change in channel resistance, where an increase in the slope indicates a rise in resistance. The variation in NDI’s slope is due to the reduced charge mobility during stretching, which increases resistance. Additionally, tensile stress may cause electrode cracking and width reduction, further amplifying the slope and making the change in the perpendicular direction more pronounced. In contrast, mAB73 shows minimal differences in charge mobility and electrode degradation, resulting in similar slopes in both directions. Morphological and resistance analyses highlight that introducing soft segments helps support electrodes under strain, reducing damage, and maintaining stable resistance behavior.

**Figure 7:**
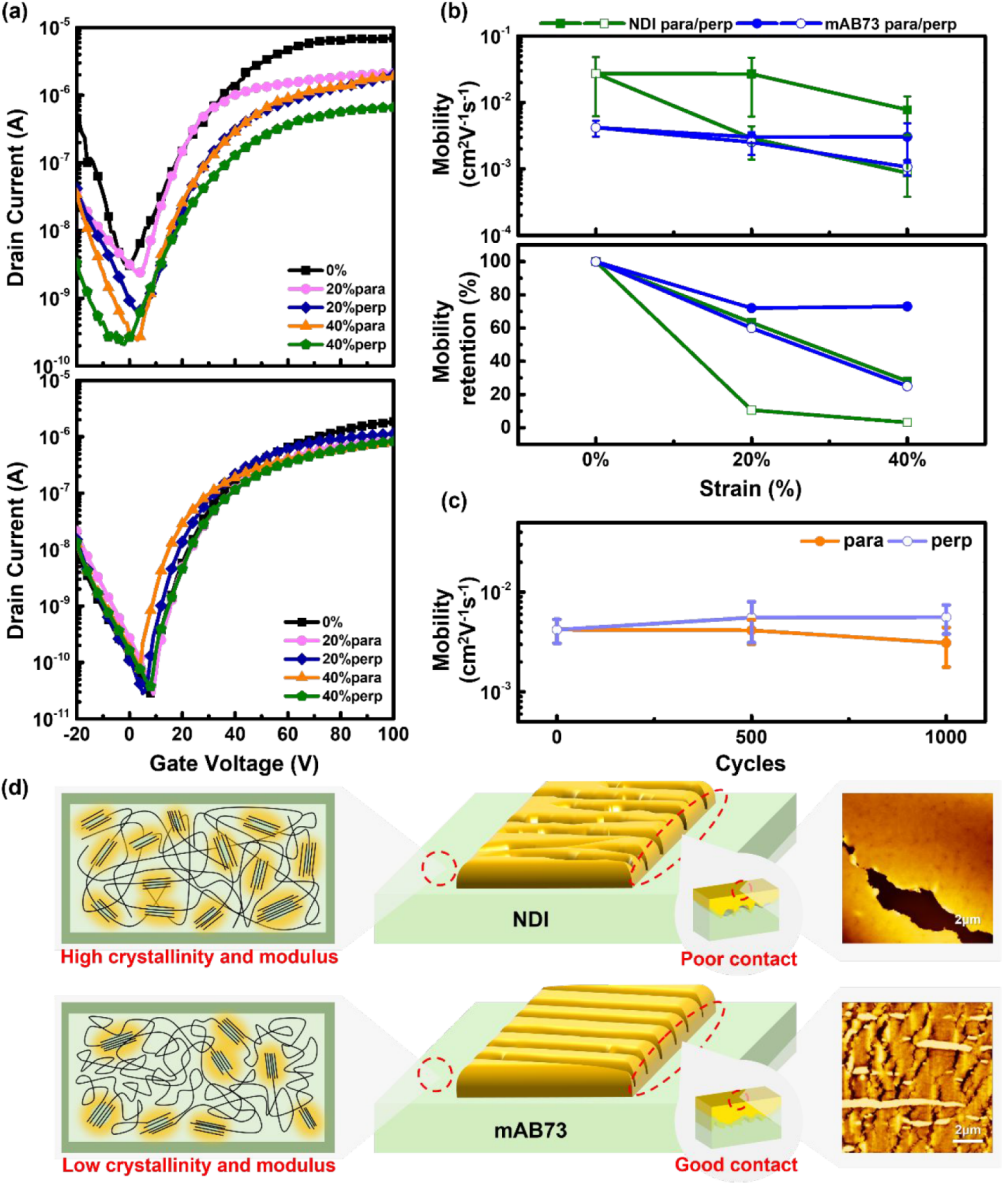
(a) OFETs transfer curves for NDI (top) and mAB73 (bottom) films with microcracked gold as contact electrodes at different strain levels. (b) Correlation of mobility (top) and mobility retention (bottom) under different strain ratios of the polymer films with microcracked gold. (c) Correlation between mobility and the stretch–release cycles for the polymer films with a strain level of 40%. (d) Schematic diagram illustrating the contact electrode cracking on polymer films and polymer crystalline structure and their influence on the mobility–stretchability properties of intrinsically stretchable OFET devices. The AFM topographies of the damaged (NDI) microcracked gold (mAB73) films are displayed on the right-hand side of the scheme.

In the integration of multi-BCPs with microcracked gold to form intrinsically stretchable OFET devices, the performance is assessed through transfer curves in Figure 7a. Here, the device current of NDI decreases as the strain increases, while mAB73 shows a more stable performance. Calculations of mobility and mobility retention are shown in Figure 7b, with other parameters compiled in Table S9. For NDI at 0%, 20%, and 40% strains, the electron mobilities in the parallel and perpendicular directions are 0.027, 0.017, 0.0029, and 0.00078, 0.00087 cm^2^ V^–1^ s^–1^, respectively. For mAB73, the electron mobilities are 0.0042, 0.003, 0.0025, and 0.0011 cm^2^ V^–1^ s^–1^. The mobility retentions for NDI at 20% and 40% strain are (63.3, 10.6)% and (28.5, 3.2)% in the parallel and perpendicular directions, respectively, while mAB73 are (71.9, 60.1)% and (72.7, 25.4)%, respectively. The mobility of NDI significantly decreases under strains while mAB73 remains stable. Figure 7c and Table S10 present the cyclic stretch-release test of mAB73, demonstrating its reversibility. The strain was set at 40% based on the rDoC performance, as the electrical properties at 40% and 0% strain are similar in parallel and perpendicular directions, with minimal variation between 20% and 40%. The graph shows a gradual decrease in the parallel direction, while the perpendicular direction exhibits the opposite trend. Morphological analysis indicates that the mAB73 film remains intact without damage, ensuring that the cyclic test is unmarred. Additionally, both the electrode resistance and contact resistance exhibit minimal changes before and after stretching. Furthermore, the electrode resistance remains stable even after stretch–release cycle tests, as shown in Figure S22. This suggests that the perpendicular direction experiences compression during stretching, leading to changes in the crystalline orientation. GIWAXS data confirm that the crystalline arrangement transitions toward an edge-on orientation in the perpendicular direction after stretching. With repeated stretch–release cycles, this gradual reorientation contributes to the slow increase in charge carrier mobility. The cyclic test results confirm that mAB73 maintains stable electrical properties after multiple stretch–release cycles, demonstrating excellent mechanical durability. Figure 7d illustrates the AFM morphology after stretching, where the electrodes of the NDI exhibit extensive damage. This is attributed to the rigid polymer structure of NDI, which causes its crystalline regions to collapse under stress, leading to cracks in the film and reduced adhesion to the electrodes. In contrast, mAB73 contains flexible segments that prevent damage to the crystalline regions, maintaining the film’s integrity and electrodes due to the microcracked gold with infiltrated gold nanoparticles to the rubbery channel surface. Consequently, mAB73 demonstrates superior adhesion to the electrodes under strains. The above concept is schematically illustrated in Figure 7d. To gain insight into the mobility–stretchability relationship of the integrated device, factors, such as (i) electrode resistance, (ii) contact resistance, and (iii) channel mobility and channel resistance, play critical roles in the system combining the polymer semiconductor and microcracked gold: (i) NDI and mAB73 show significant differences in gold electrode resistance due to the infiltrated gold nanoparticles. (ii) Due to their different interfacial contacts, contact resistance for NDI and mAB73 increases with strain by approximately 10–100 and 1–3 times, respectively. (iii) Regarding polymer charge mobility performance, NDI’s mobility decreases by 10–20%, while mAB73 retains consistent performance with strain-insensitive mobility due to its stable crystallographic and morphological properties. From the TLM slope, it can be observed that the channel resistance of NDI undergoes significant changes, whereas mAB73 exhibits minor variations. The channel resistance changes for NDI range from 2 to 20 times, while for mAB73, it ranges from 1 to 3 times. The variation in channel resistance corresponds well with changes in carrier mobility. Based on the above analysis, under integrated device systems at 40% strain, the charge mobility of NDI decreases by 71% and 97% in the parallel and perpendicular strain directions, respectively, while mAB73 decreases by 27% and 75%, respectively. Overall device performance indicates that contact resistance has the most substantial impact, followed by channel mobility and channel resistance, with electrode resistance having a minimal influence. Analysis of the integrated stretchable device system shows that multi-BCPs exhibit high stability with minimal variations in resistance and charge mobility. Thus, multi-BCPs are demonstrated to be materials with a perspective on utilizing microcracked contact electrodes for stretchable devices.

## Conclusion

We successfully synthesized novel N-type multi-BCPs composed of rigid PNDI2T and flexible PIB segments as polymer semiconductors for the first time. The characterization of multi-BCP results indicates a high impact of introducing soft segments into conjugated polymers on surface morphology, crystallinity, and electrical properties under strain. The multi-BCPs exhibit excellent stretchability, with no cracks observed even at 100% strain. Among the materials, mAB60, which contains more soft segments than mAB73, shows a lower elastic modulus, confirming that incorporating PIB significantly enhances the polymer’s extensibility. For the performance of OFET devices, introducing soft segments leads to decreased charge mobility. The measured mobilities for NDI, mAB73, and mAB60 are 0.091, 0.017, and 5.6 × 10^–4^ cm^2^ V^–1^ s^–1^, respectively, consistent with previous studies showing that increased disordered regions in polymers reduce charge transport. However, incorporating soft segments positively affects mobility retention under strain. mAB73’s mobility is insensitive to different strain levels spanning the range of 0–100%, supported by a gradual increase in the rDOC. In contrast, mAB60 shows deteriorated mobility retention under strain due to microphase separation and disorder induced by tensile stress. Thus, the appropriate incorporation of soft segments in multi-BCPs is critical for optimizing stretchability and charge transport properties. At the end of this study, an integrated stretchable device was demonstrated to explore the performance of multi-BCPs with microcracked gold. Multi-BCPs with soft segments demonstrate superior electrode integrity and suppress contact and electrode resistance increases. mAB73 achieves a mobility retention of ∼70% under 20% and 40% strains in the horizontal direction, indicating strong interfacial adhesion between the mAB73 film and microcracked gold. Additionally, mAB73 exhibits significantly greater stability than NDI in integrated systems where the polymer and electrode are simultaneously stretched.These findings highlight the influence of soft-segment incorporation on mechanical properties, crystallinity, and electrical performance. Multi-BCPs exhibit microcracked gold with infiltrated gold nanoparticles on the rubbery channel surface, thereby achieving an outstanding mobility–stretchability relationship in the integrated and intrinsically stretchable OFET device.

## Supporting Information Available

Synthesis and chemical structure characterizations of the multi-BCPs; optical absorption and electrochemical spectra of the multi-BCP films; OM, AFM, GIWAXS, and GISAXS images, crystallographic parameters, and transfer characteristics of the stretched polymer films; contact resistance measurements of the stretched polymer films with microcracked gold as contact electrodes (PDF)
