# Supplementary material for: Conjugated Multiblock Copolymers and Microcracked Gold Electrodes Applied for the Intrinsically Stretchable Field-Effect Transistor
Source: ACS Appl Mater Interfaces. 2025 Mar 27;17(14):21521–35. doi: 10.1021/acsami.5c00047 (PMC11986895; doi:10.1021/acsami.5c00047)
Supplement: Supplementary file 1 — am5c00047_si_001.pdf [file am5c00047_si_001.pdf]

Supporting Information

Conjugated Multiblock Copolymers and Mircrocracked Gold Electrodes Applied for the Intrinsically Stretchable Field-Effect Transistor

Yu-Chun Huang [a], Shuto Yamamoto [b], Jung-Yao Chen [c], Chun-Jen Su [d], U-Ser Jeng [d,e], Tomoya Higashihara [b], Yan-Cheng Lin [a,f*]*

[a] Department of Chemical Engineering, National Cheng Kung University, Tainan 70101, Taiwan.

[b] Department of Organic Materials Science, Graduate School of Organic Materials Science, Yamagata University, 4-3-16 Jonan, Yonezawa, Yamagata 992-8510, Japan.

[c] Department of Photonics, National Cheng Kung University, Tainan City 70101, Taiwan.

[d] National Synchrotron Radiation Research Center, Hsinchu 300092, Taiwan.

[e] Department of Chemical Engineering & College of Semiconductor Research, National Tsing Hua University, Hsinchu 300044, Taiwan.

[f] Advanced Research Center for Green Materials Science and Technology, National Taiwan University, Taipei 10617, Taiwan.

[*] E-mail: thigashihara@yz.yamagata-u.ac.jp (T. Higashihara); ycl@gs.ncku.edu.tw (Y.-C. Lin)

Synthesis of α,ω-chain-end-functionalized PIB with thien-2-yl group (T-PIB-T).

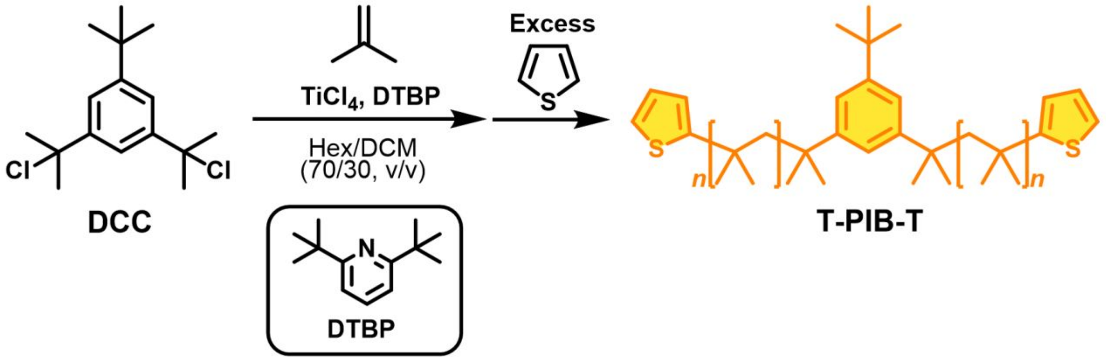

Scheme S1. Synthetic route for T-PIB-T.

In a glove box under a nitrogen atmosphere, the Schlenk flask was charged with 5-*tert*-butyl-1,3-bis(1-chloro-1-methylethyl)benzene (DCC, 0.100 g, 0.350 mmol), 2,6-di-*tert*-butylpyridine (DTBP, 0.0900 g, 0.470 mmol), hexane (34 mL, deoxidized), and DCM (16.7 mL, deoxidized). The sealed Schlenk flask was taken out from a glovebox and then cooled down to –78 °C using a dry ice/acetone bath. Isobutene (2.85 mL at –78 °C, 30.0 mmol) was added to this solution. The polymerization was initiated by adding titanium(IV) chloride solution in hexane (10 mL, deoxidized)/DCM (4.3 mL, deoxidized) (70/30, v/v), which was prepared in a syringe vial inside the glove box in advance, and the solution was stirred for 1.5 h at –78 °C. To the reaction mixture, a thiophene (11.8 g, 140 mmol) solution in hexane (5.0 mL, deoxidized)/DCM (2.1 mL, deoxidized) (70/30, v/v), which was prepared in a syringe vial inside the glove box in advance, was added. The solution was stirred at –78 °C for another 4.5 h. The reaction was quenched with methanol (–78 °C), and the resulting solution was poured into a methanol/ammonia hydroxide aqueous solution (90/10, v/v). The mixture was then washed with water/isopropanol/NaCl (77.5/15/7.5, v/v/w) and water twice for each. The polymer was reprecipitated with hexane/methanol to afford T-PIB-T as a colorless liquid (1.78 g, 98%). M_n (SEC) = 7,300, M_n (^{1}H NMR) = 6,500, M_w/M_n (SEC)= 1.12.

^{1}H NMR (400 MHz, $CDCl_3$, 25 ℃, **Figure S1**) δ (ppm) : 7.17 (s, 3H), 7.11–7.09 (m, 2H), 6.88 (dd, J = 5.0, 2H), 6.79 (dd, 2H), 1.69 (s, 0H), 1.41–1.37 (m, 212H), 1.11–1.02 (m, 649H).

Synthesis of α,ω-chain-end-functionalized PIB with 5-bromothien-2-yl group (BrT-PIB-TBr).

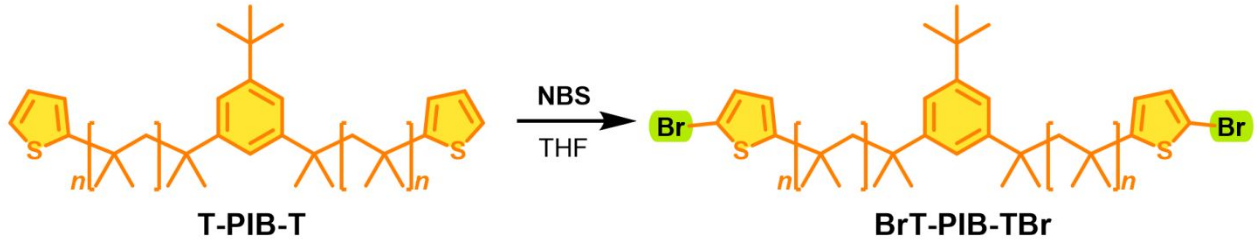

Scheme S2. Synthetic route for BrT-PIB-TBr.

Into a 20 mL two-necked flask, T-PIB-T (0.500 g, 0.0780 mmol) and tetrahydrofuran (THF) (9.15 mL) were added. After cooling the solution to 0 °C using an ice bath, *N*-bromosuccinimide (NBS, 0.280 g, 1.56 mmol) was added to the flask. The mixture was stirred at room temperature overnight. After the reaction, the solution was rotary-evaporated, and the residue was poured into hexane to precipitate the byproduct. After filtration, the filtrate was concentrated, and methanol was added to precipitate the polymer. The resulting polymer was reprecipitated with hexane/methanol to afford BrT-PIB-TBr as a colorless oil (0.340 g, 66%). M_n (SEC) = 7,200, M_n (^{1}H NMR) = 6,600, M_w/M_n (SEC)= 1.11.

^{1}H NMR (400 MHz, $CDCl_3$, 25 ℃, **Figure S2**) δ (ppm) : 7.16 (s, 3H), 6.81 (t, 2H), 6.54 (t, 2H), 1.82 (s, 4H), 1.77 (d, 4H), 1.41–1.34 (m, 239H), 1.10–1.01 (m, 667H).

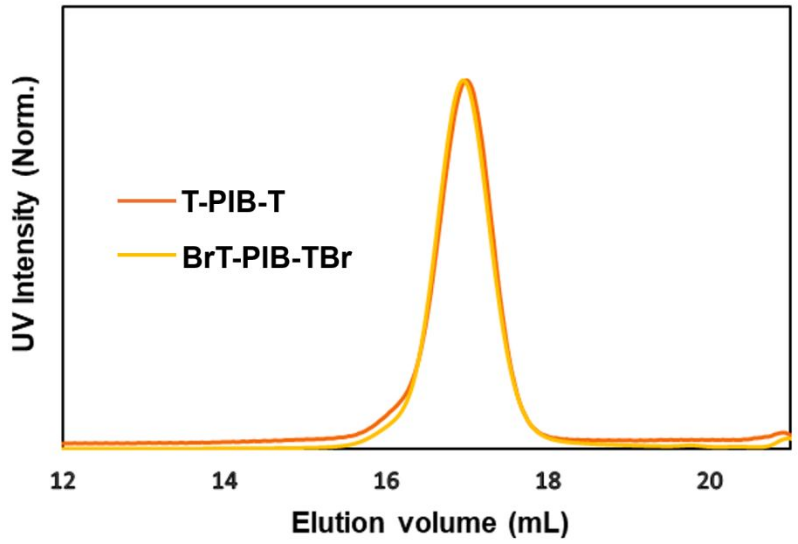

Figure S1. SEC UV traces of T-PIB-T and BrT-PIB-TBr.

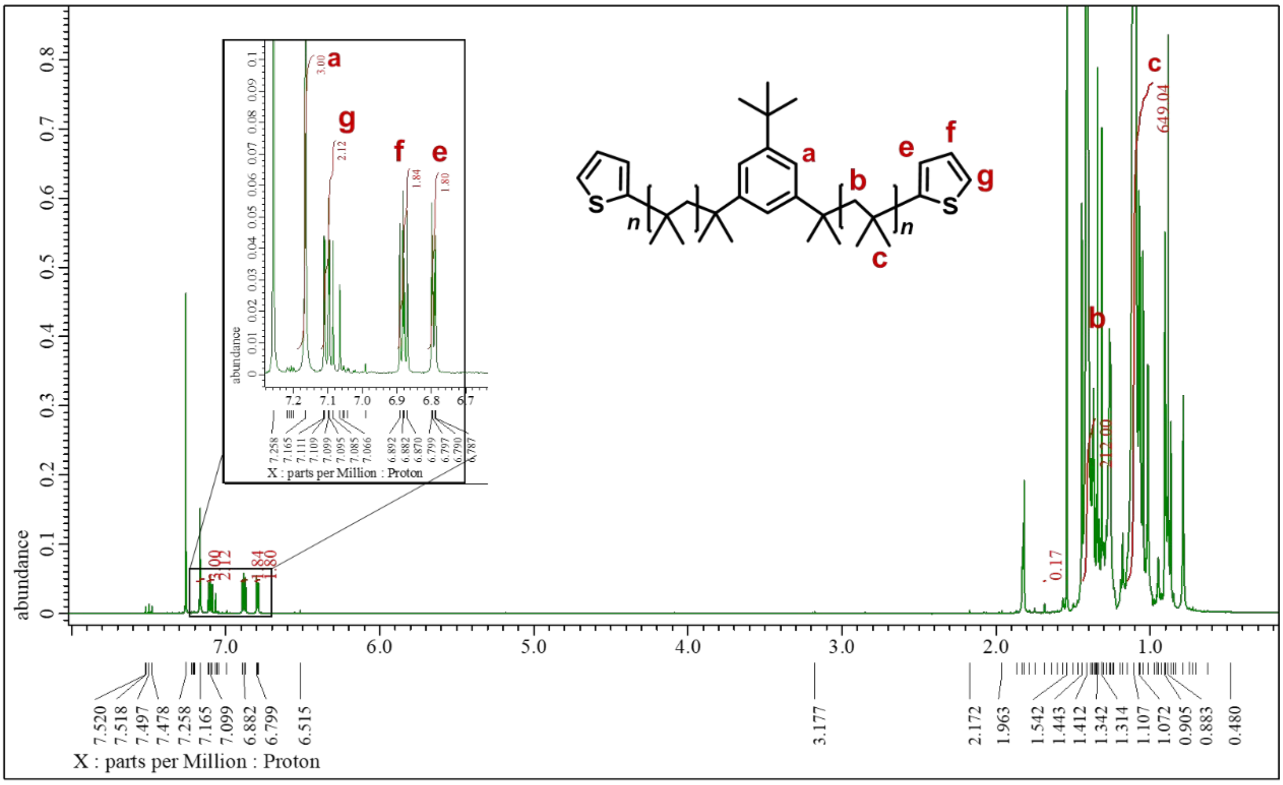

Figure S2. ^{1}H NMR spectrum of T-PIB-T.

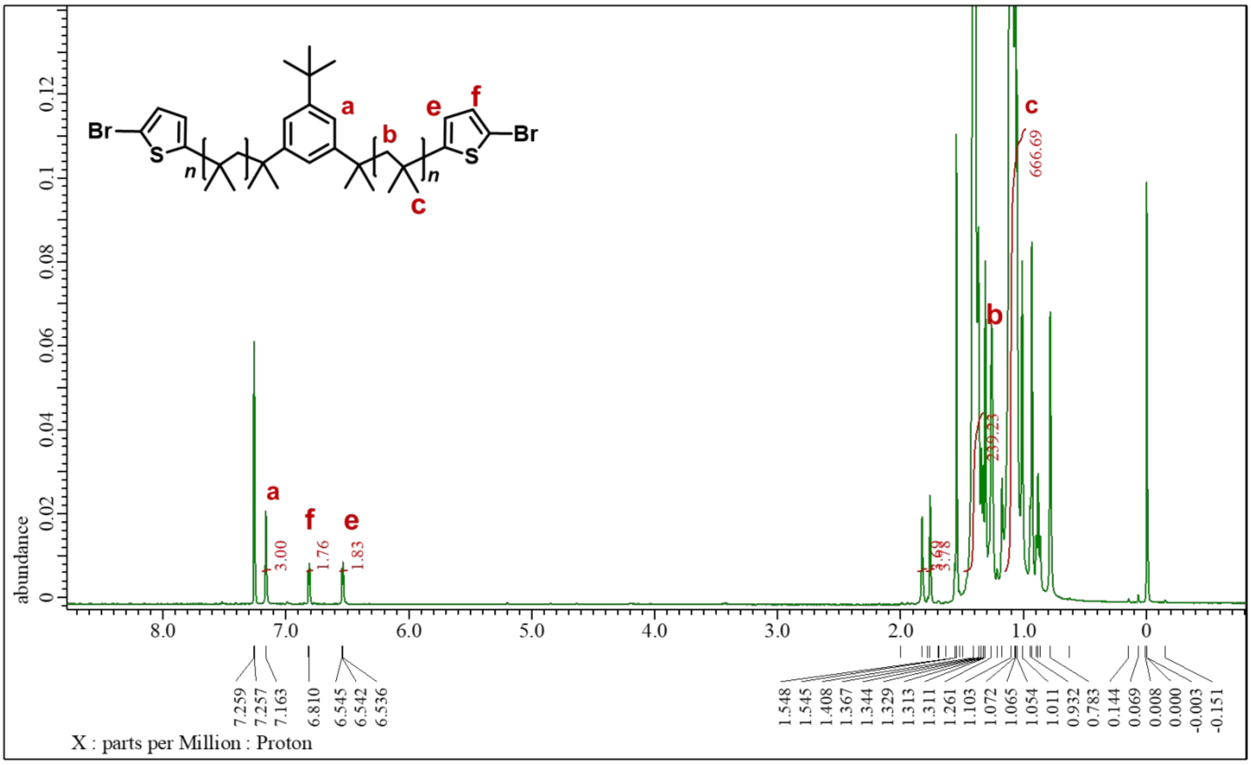

Figure S3. ^{1}H NMR spectrum of BrT-PIB-TBr.

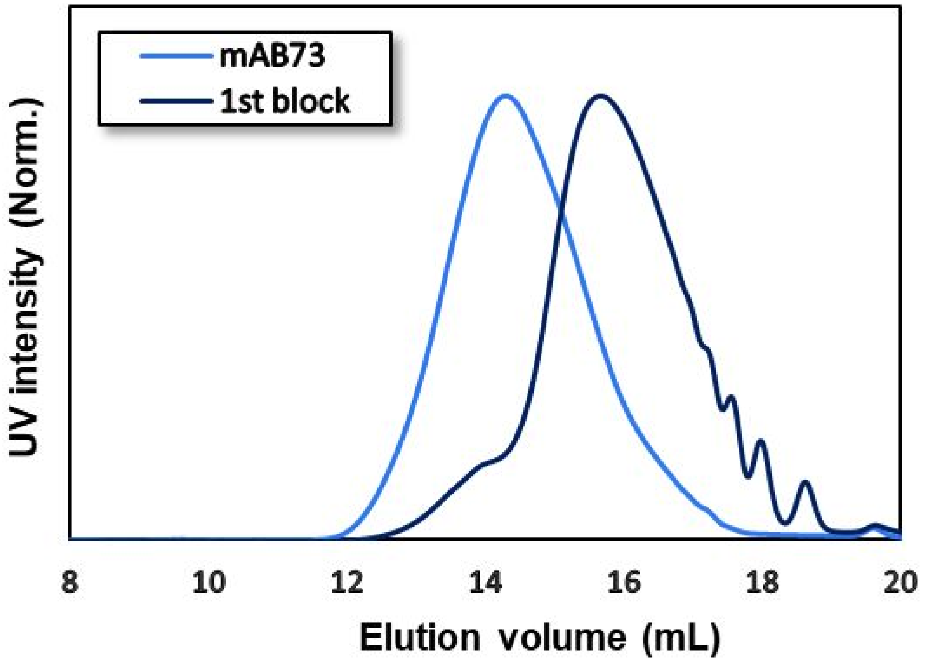

Figure S4. SEC UV traces of 1st block and mAB73.

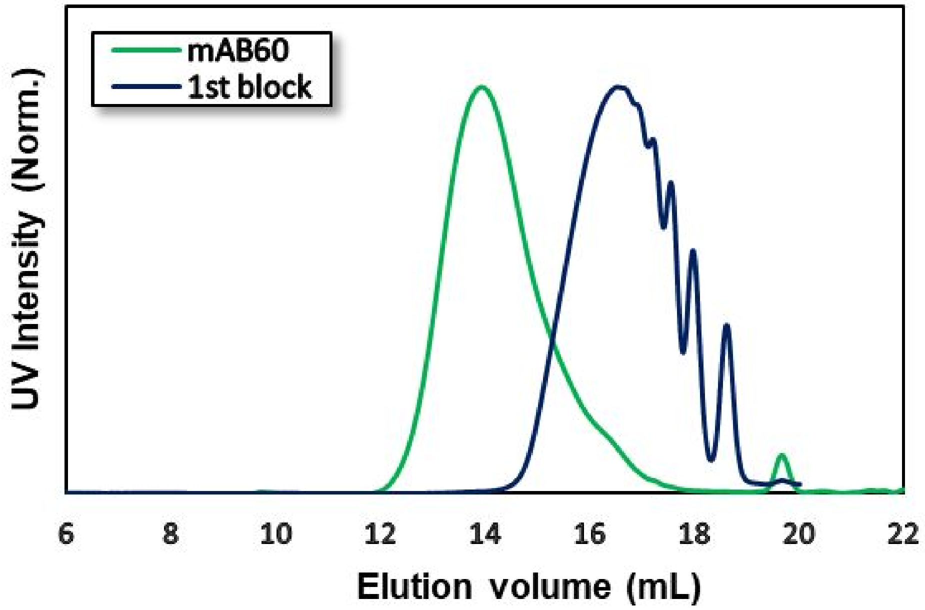

Figure S5. SEC UV traces of 1st block and mAB60.

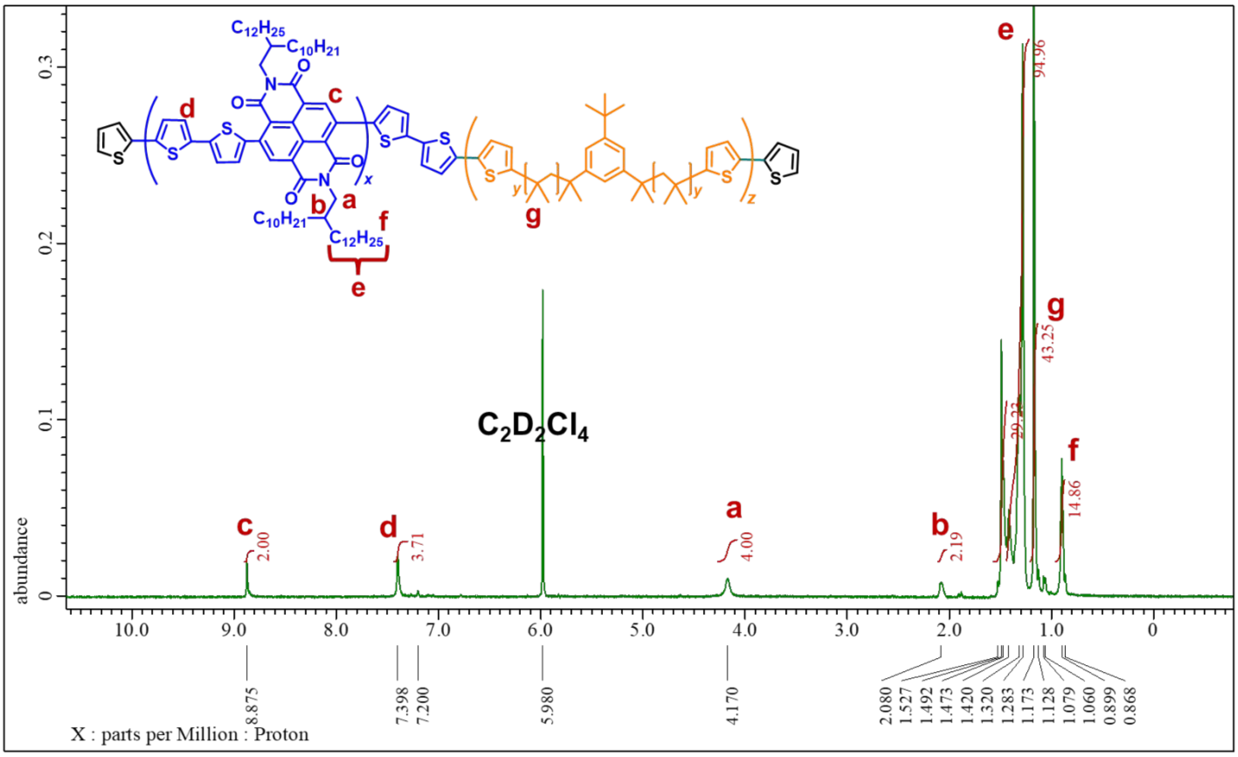

Figure S6. ^{1}H NMR spectrum of mAB73.

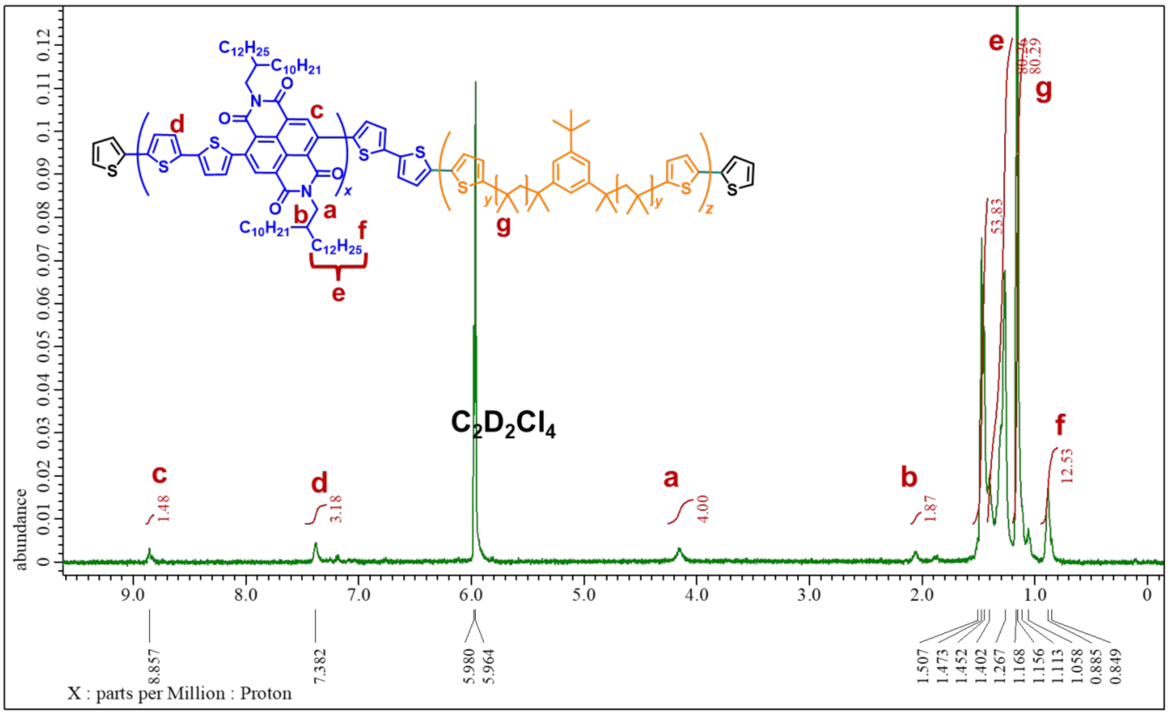

Figure S7. ^{1}H NMR spectrum of mAB60.

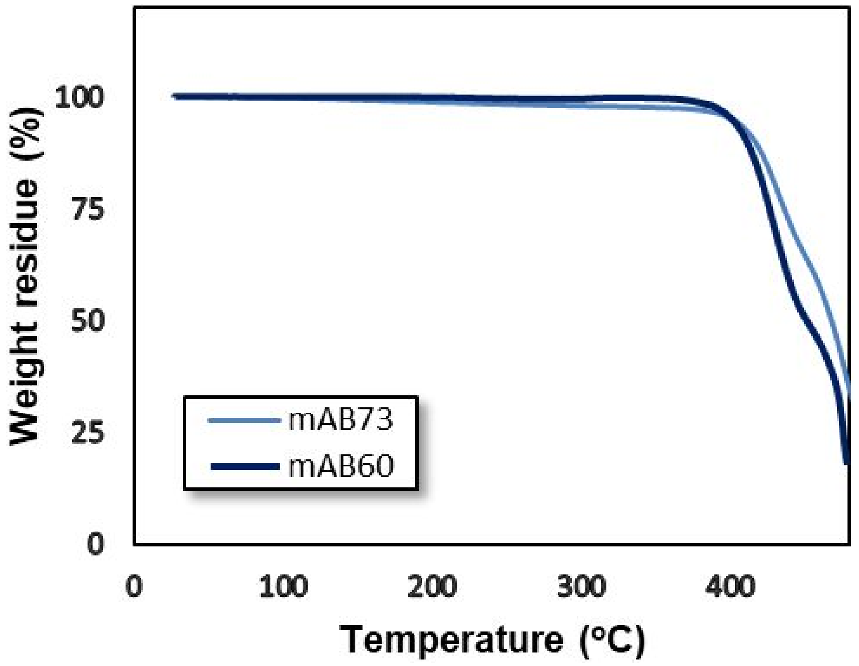

Figure S8. TGA thermograms of mAB73 and mAB60 at a ramping rate of 10 °C/min in nitrogen.

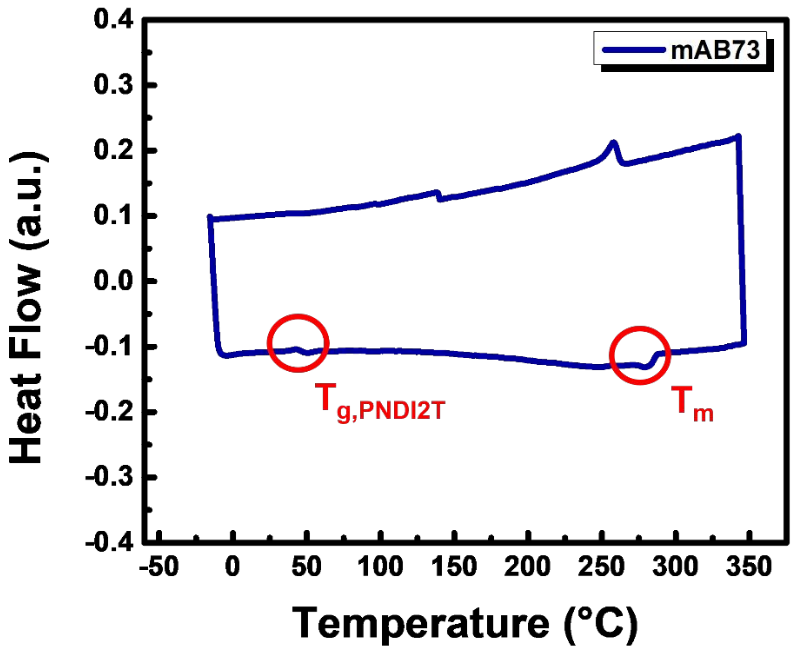

Figure S9. DSC thermograms of mAB73 at a ramping rate of 10 °C/min in nitrogen (1st cooling and 2nd heating scans).

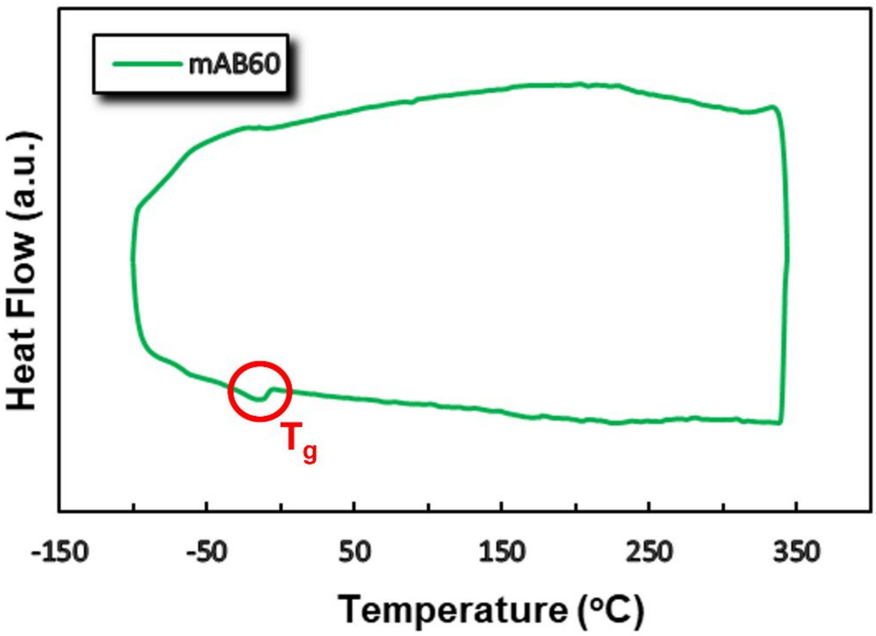

Figure S10. DSC thermograms of mAB60 at a ramping rate of 10 °C/min in nitrogen (1st cooling and 2nd heating scans).

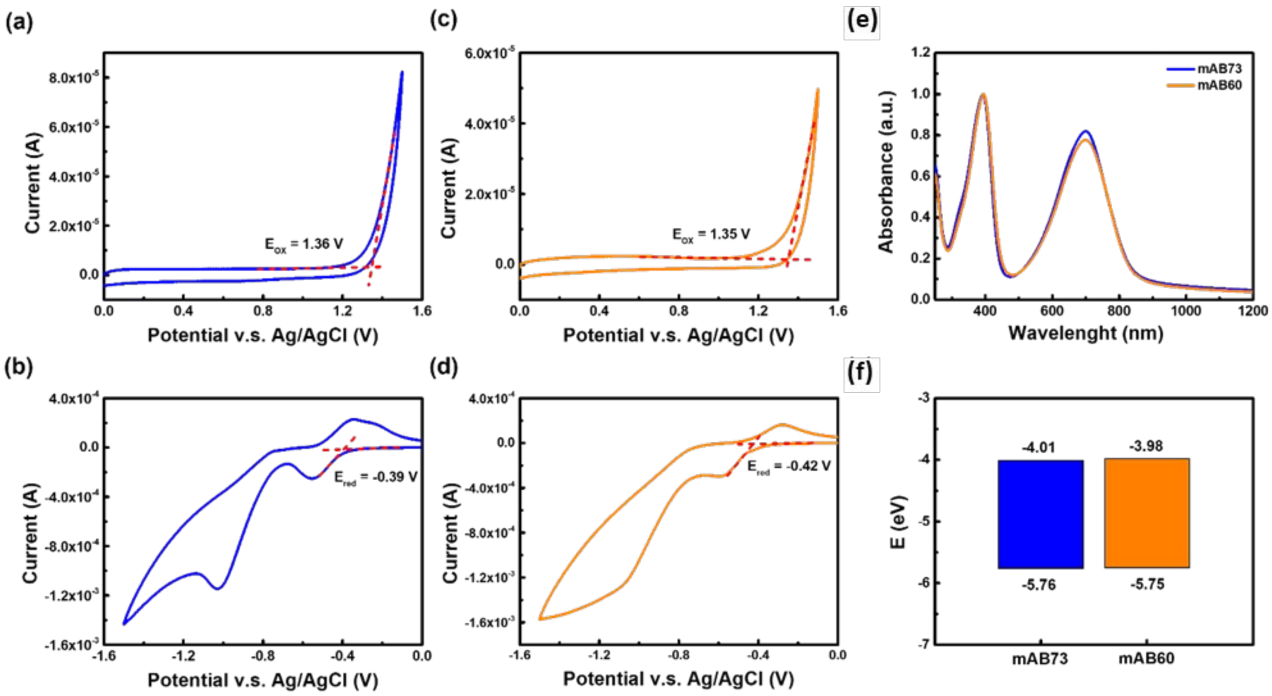

Figure S11. CV profiles showing (a, c) oxidation and (b, d) reduction sections for (a, b) mAB73 and (c, d) mAB60 thin films. (e) UV-vis absorption spectra of the polymer films. (f) The frontier energy levels of the polymer films studied.

Table S1. Optical and electrochemical parameters of the multi-BCPs studied. Note that the HOMO level was calculated from the CV oxidative potential (E_{ox}), and the LUMO level was calculated from the CV reductive potential (E_{red}).

	λ_{max} (nm)	E_{ox} (V)	E_{red} (V)	HOMO (eV)	LOMO (eV)	$E_{g,CV}$ (eV)	$E_{g,opt}$ (eV)
**mAB73**	852.0	1.36	–0.39	–5.76	–4.01	1.75	1.46
**mAB60**	851.4	1.35	–0.42	–5.75	–3.98	1.77	1.47

Note that $E_{g,CV}$ and $E_{g,opt}$ represent the bandgap derived from the electrochemical or optical measurements.

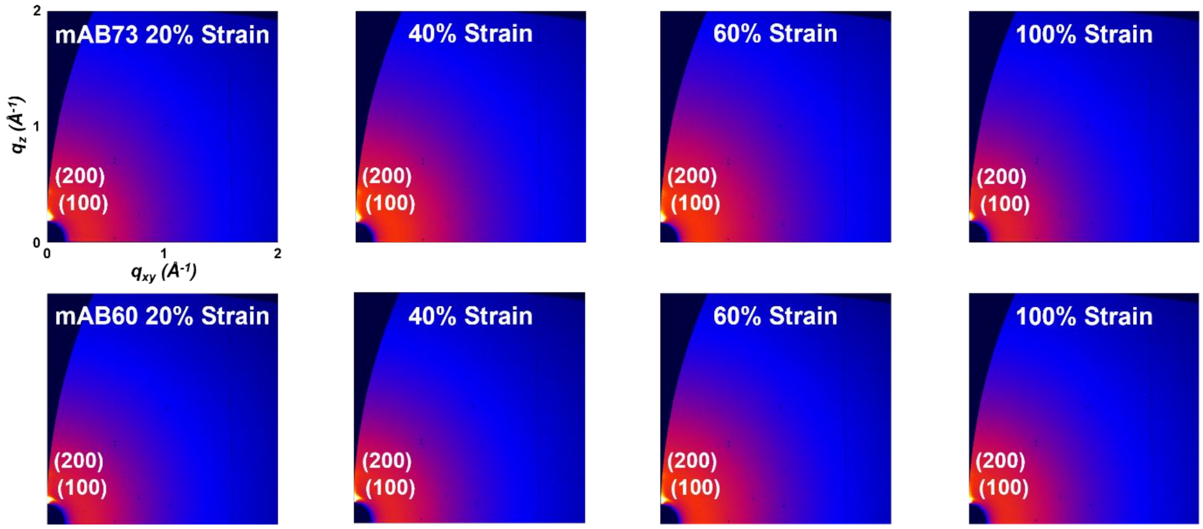

Figure S12. 2D GIWAXS patterns of the transferred/stretched mAB73 (top) and mAB60 (bottom) polymer films at different strain ratios with the stretching direction perpendicular to the incident beam.

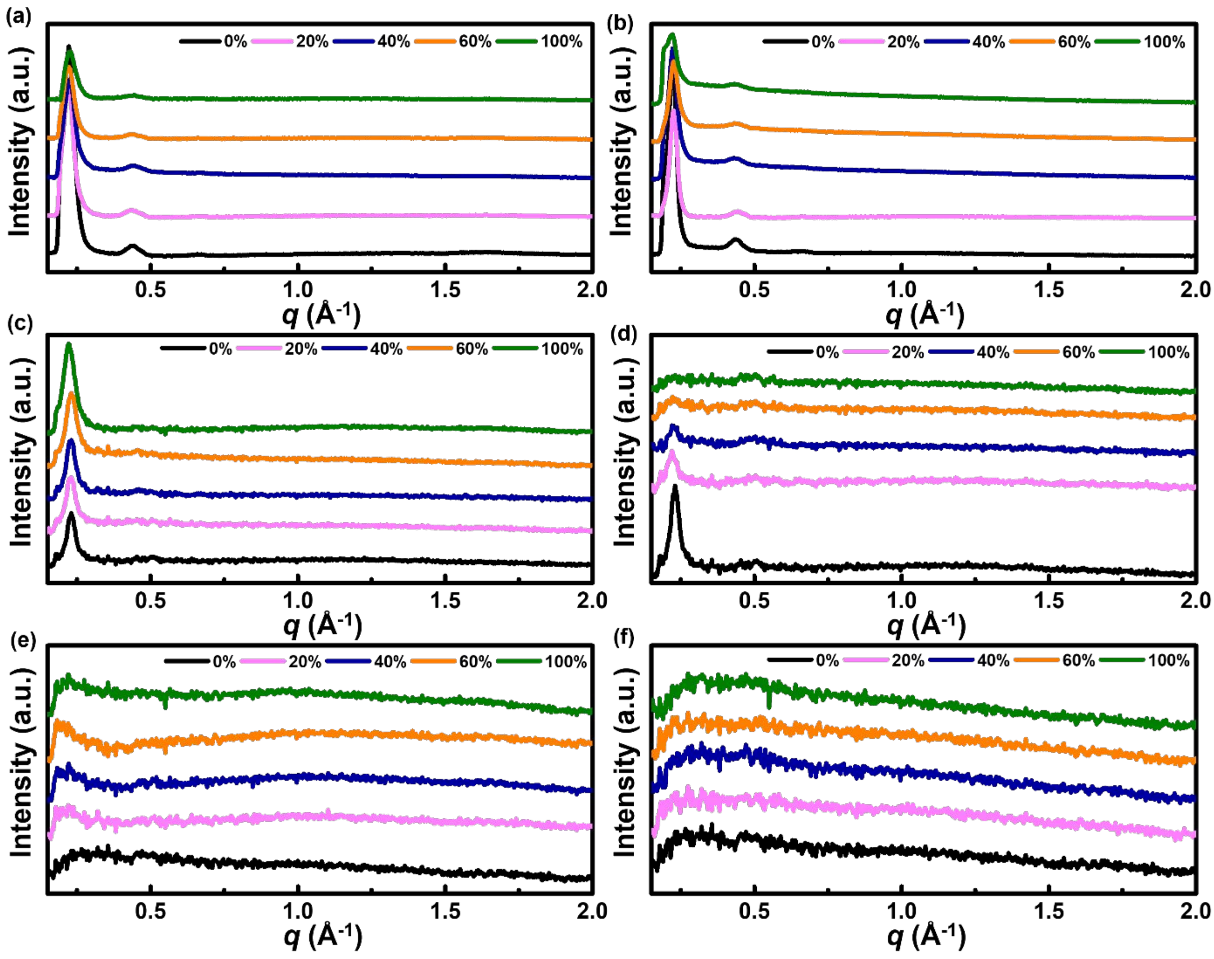

Figure S13. 1D GIWAXS profile along the OOP direction of the transferred/stretched (a) mAB73 and (b) mAB60 polymer films at different strain ratios with the stretching direction perpendicular to the incident beam. 1D GIWAX profile along the IP direction of the transferred/stretched (c, d) mAB73 and (e, f) mAB60 polymer films at different strain ratios with the stretching direction (c, e) parallel and (d, f) perpendicular to the incident beam.

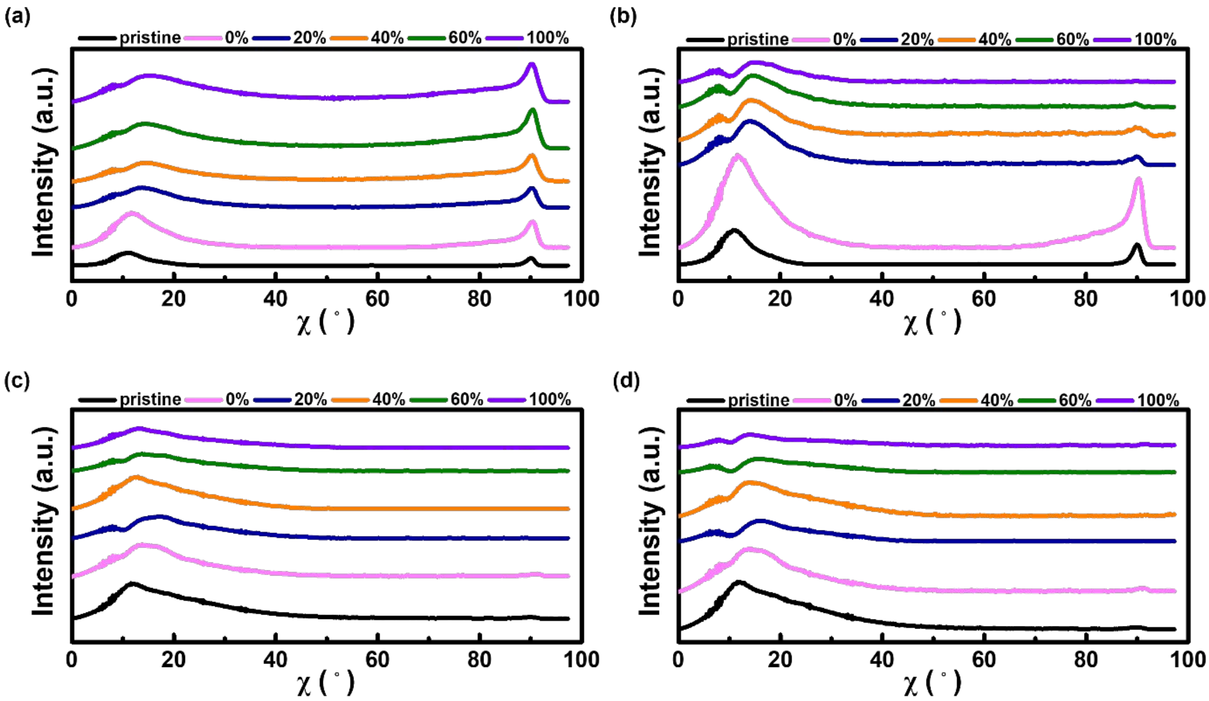

Figure S14. GIWAXS Pole figure for the (100) diffraction peaks of (a, b) mAB73 and (c, d) mAB60 polymer films at different strain ratios with the stretching direction (a, c) parallel and (b, d) perpendicular to the incident beam.

Table S2. GIWAXS crystallographic parameters, including the OOP lamellar stacking distance (d_{100}), coherence length (L_c), and paracrystalline disorder (g), for the transferred/stretched polymer films at varied strain levels.

		mAB73			mAB60		
**Strain (%)**		d_{100} (Å)	L_c (Å)	g	d_{100} (Å)	L_c (Å)	g
**Pristine**		28.3	118.2	0.185	29.2	133.6	0.177
**0%**		28.2	112.1	0.190	28.5	138.5	0.172
**20%**	‖	28.0	104.5	0.196	28.2	134.9	0.173
	⊥	28.2	111.2	0.191	28.0	134.5	0.173
**40%**	‖	27.8	103.4	0.196	28.8	137.3	0.173
	⊥	27.3	90.5	0.208	28.1	129.9	0.176
**60%**	‖	27.7	94.9	0.204	28.6	124.4	0.182
	⊥	27.7	99.1	0.200	27.6	124.5	0.178
**100%**	‖	28.1	108.7	0.192	29.2	124.3	0.183
	⊥	27.1	99.0	0.198	28.3	118.8	0.185

Table S3. GIWAXS crystallographic parameters, including the IP lamellar stacking distance (d_{100}), coherence length (L_c), and paracrystalline disorder (g), of the transferred/stretched polymer films at varied strain levels.

		mAB73			mAB60		
**Strain (%)**		d_{100} (Å)	L_c (Å)	g	d_{100} (Å)	L_c (Å)	g
**Pristine**		28.0	128.2	0.177	--	--	--
**0%**		27.3	144.6	0.164	--	--	--
**20%**	‖	27.3	130.6	0.173	--	--	--
	⊥	28.4	139.6	0.171	--	--	--
**40%**	‖	27.3	139.6	0.167	--	--	--
	⊥	27.7	169.3	0.153	--	--	--
**60%**	‖	27.1	118.3	0.181	--	--	--
	⊥	27.9	61.0	0.256	--	--	--
**100%**	‖	28.2	108.7	0.193	--	--	--
	⊥	--	--	--	--	--	--

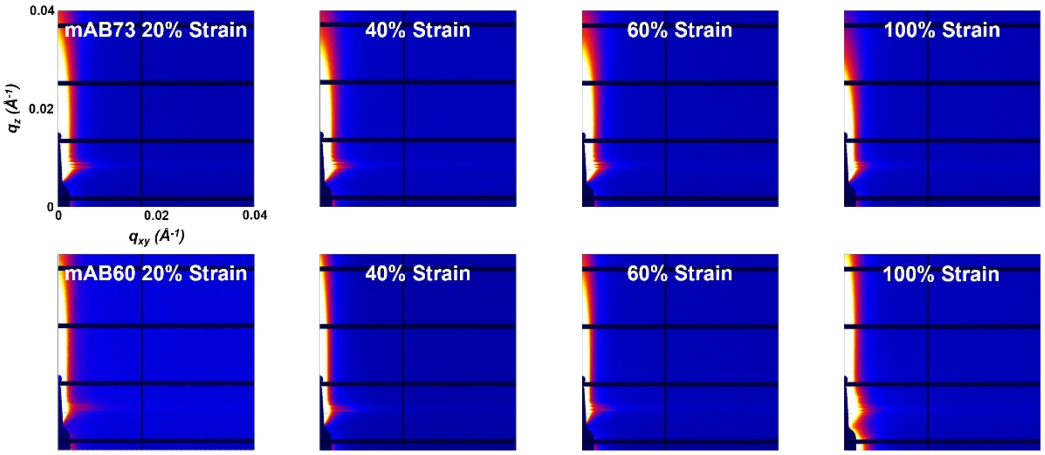

Figure S15. 2D GISAXS patterns of the transferred/stretched mAB73 (top) and mAB60 (bottom) films at different strain ratios with the stretching direction perpendicular to the incident beam.

Table S4. GISAXS domain distance (*d*) of the multi-BCP films.

		**mAB73**	**mAB60**
**Strain (%)**		***d*** **(Å)**	***d*** **(Å)**
**Pristine**		964	920
**0%**		--	899
**20%**	‖	--	858
	⊥	--	955
**40%**	‖	--	853
	⊥	--	1091
**60%**	‖	--	801
	⊥	--	1149
**100%**	‖	--	731
	⊥	--	--

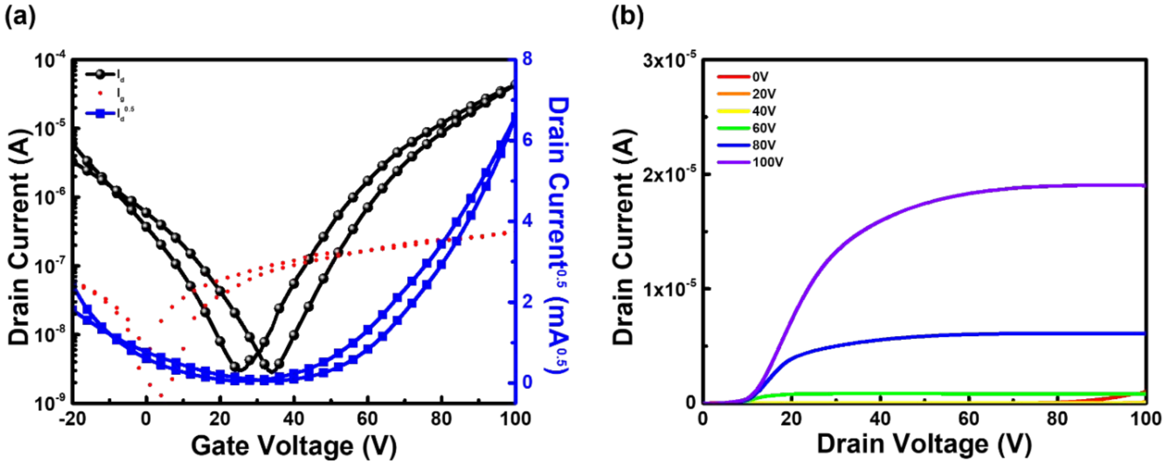

Figure S16. OFET (a) transfer and (b) output curves for regular NDI films.

Table S5. OFETs device parameters, including the electron mobility (μ_e), current contrast (I_{on}/I_{off}), and threshold voltage (V_{th}), of the regular polymer films derived from three different device batches and averaged from ten units.

	μ_e (cm^2 V^{-1} s^{-1})	I_{on}/I_{off}	V_{th} (V)
**NDI**	$9.1 \times 10^{-2} \pm 3.8 \times 10^{-3}$	1×10^4	51
**mAB73**	$1.7 \times 10^{-2} \pm 1.6 \times 10^{-3}$	3×10^3	37
**mAB60**	$5.6 \times 10^{-4} \pm 1.7 \times 10^{-4}$	7×10^2	28

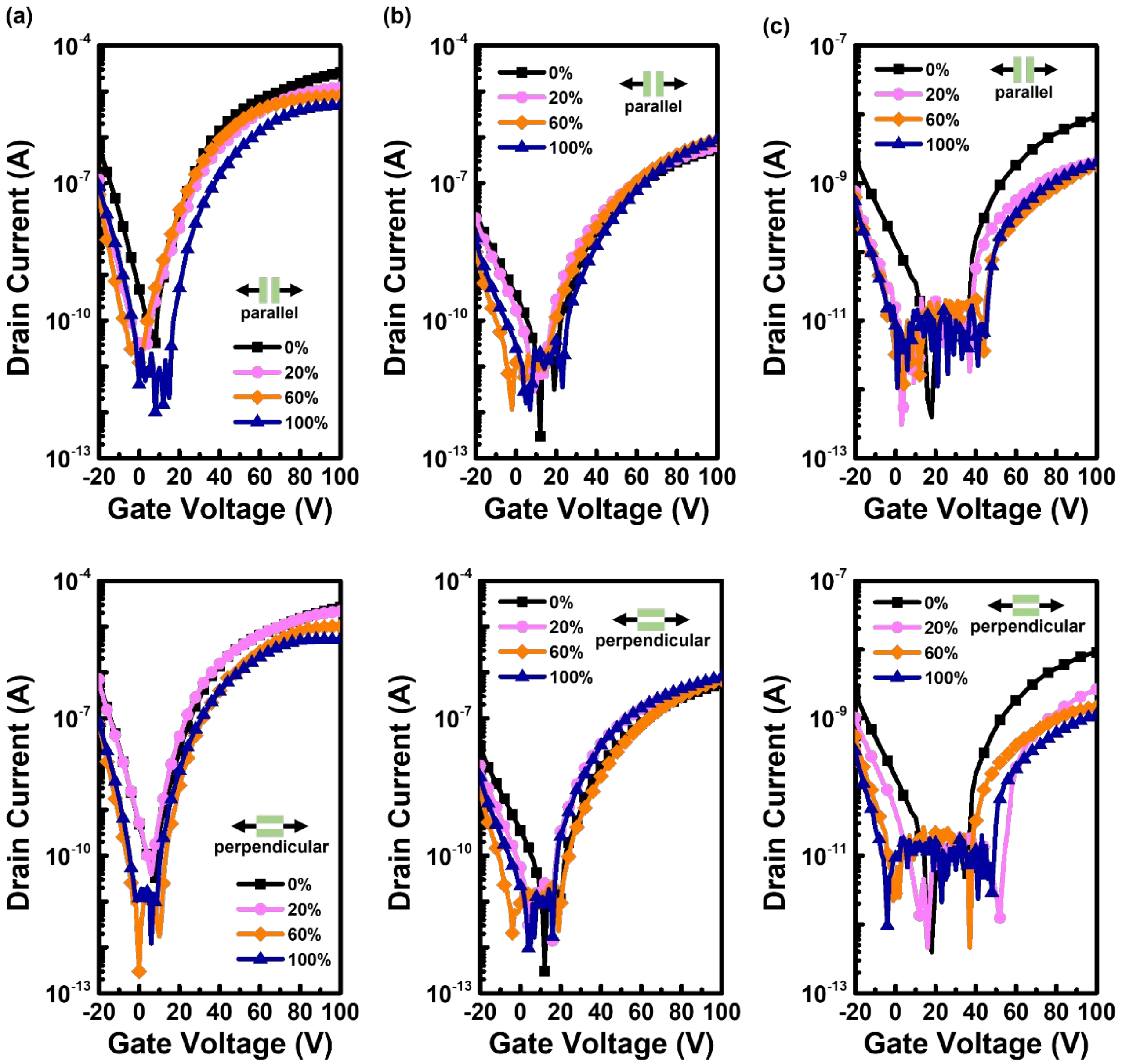

Figure S17. OFETs transfer curve for (a) NDI, (b) mAB73, and (c) mAB60 of polymer films with different strains (top) parallel or (bottom) perpendicular to the channel direction.

Table S6. OFET parameters, including the electron mobility (μ_e), current contrast (I_{on}/I_{off}), and threshold voltage (V_{th}), of NDI and multi-BCP films at different strain levels derived from three different device batches and averaged from ten units.

		NDI			mAB73			mAB60		
**Strain (%)**		**μ_e (cm^2 V^{-1} s^{-1})**	**I_{on}/I_{off}**	**V_{th} (V)**	**μ_e (cm^2 V^{-1} s^{-1})**	**I_{on}/I_{off}**	**V_{th} (V)**	**μ_e (cm^2 V^{-1} s^{-1})**	**I_{on}/I_{off}**	**V_{th} (V)**
**0%**		$3.9 \times 10^{-2} \pm 7.9 \times 10^{-3}$	5×10^6	24	$1.4 \times 10^{-3} \pm 4.2 \times 10^{-4}$	2×10^6	35	$1.9 \times 10^{-5} \pm 9.7 \times 10^{-6}$	3×10^4	34
**20%**	‖	$3.8 \times 10^{-2} \pm 5.2 \times 10^{-3}$	4×10^6	21	$1.5 \times 10^{-3} \pm 2.8 \times 10^{-4}$	3×10^5	38	$4.2 \times 10^{-6} \pm 1.3 \times 10^{-6}$	3×10^4	26
	⊥	$4.4 \times 10^{-2} \pm 4.7 \times 10^{-3}$	2×10^6	23	$1.6 \times 10^{-3} \pm 1.4 \times 10^{-4}$	1×10^7	37	$5.7 \times 10^{-6} \pm 3.0 \times 10^{-6}$	3×10^4	31
**60%**	‖	$3.0 \times 10^{-2} \pm 3.3 \times 10^{-3}$	5×10^6	26	$1.5 \times 10^{-3} \pm 8.2 \times 10^{-4}$	1×10^6	35	$4.4 \times 10^{-6} \pm 1.1 \times 10^{-6}$	8×10^3	30
	⊥	$3.5 \times 10^{-2} \pm 5.0 \times 10^{-3}$	6×10^6	20	$1.4 \times 10^{-3} \pm 7.4 \times 10^{-4}$	8×10^5	37	$3.8 \times 10^{-6} \pm 9.0 \times 10^{-7}$	4×10^3	29
**100%**	‖	$1.8 \times 10^{-2} \pm 2.1 \times 10^{-3}$	4×10^6	27	$1.5 \times 10^{-3} \pm 3.6 \times 10^{-4}$	1×10^6	41	$3.2 \times 10^{-6} \pm 1.9 \times 10^{-6}$	3×10^3	34
	⊥	$2.2\times 10^{-2} \pm 2.5 \times 10^{-3}$	5×10^6	23	$1.6 \times 10^{-3} \pm 2.7 \times 10^{-4}$	2×10^6	39	$2.7 \times 10^{-6} \pm 1.4 \times 10^{-6}$	4×10^4	34

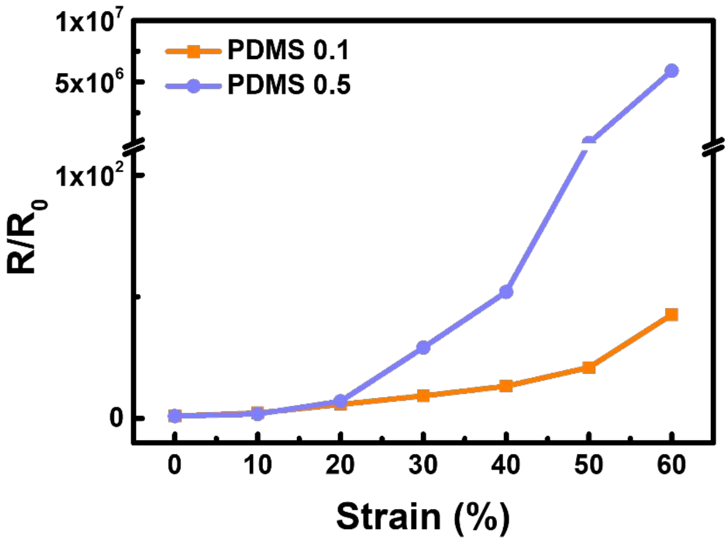

Figure S18. The electrode resistance of PDMS with different deposition rates of 0.1 and 0.5 Å/s.

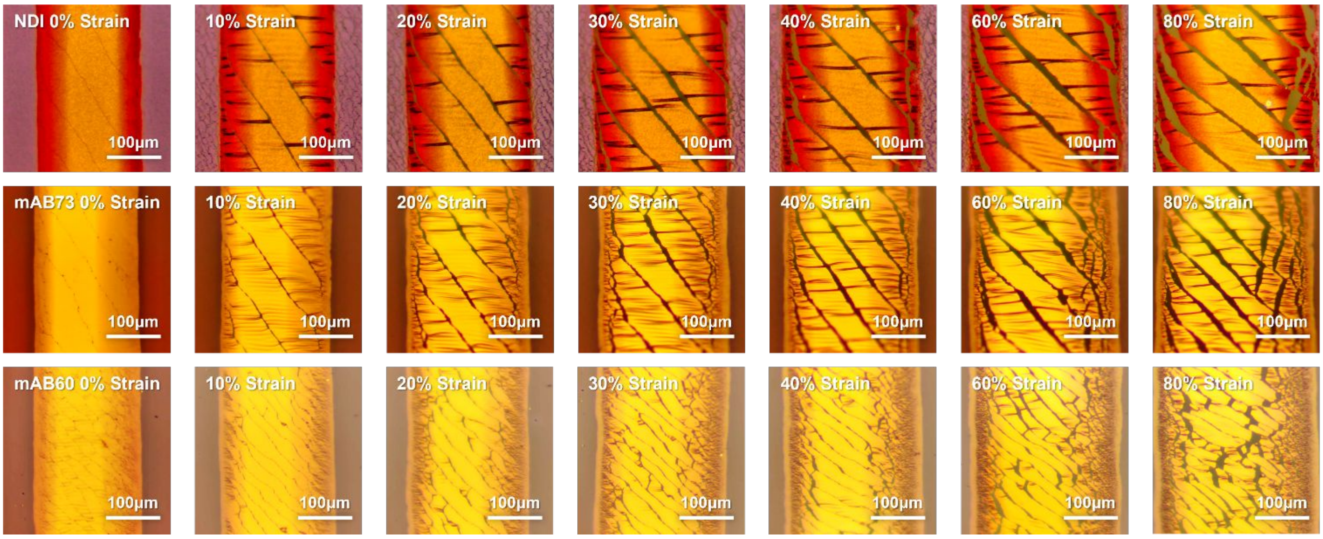

Figure S19. OM images for NDI (top), mAB73 (middle), and mAB60 (bottom) films at different strains with the stretching direction parallel to the polymer channel. Note that the parallelly stretched gold is 60 μm long and 943.8 μm wide at 20% strain and 70 μm long and 887.5 μm wide at 40% strain.

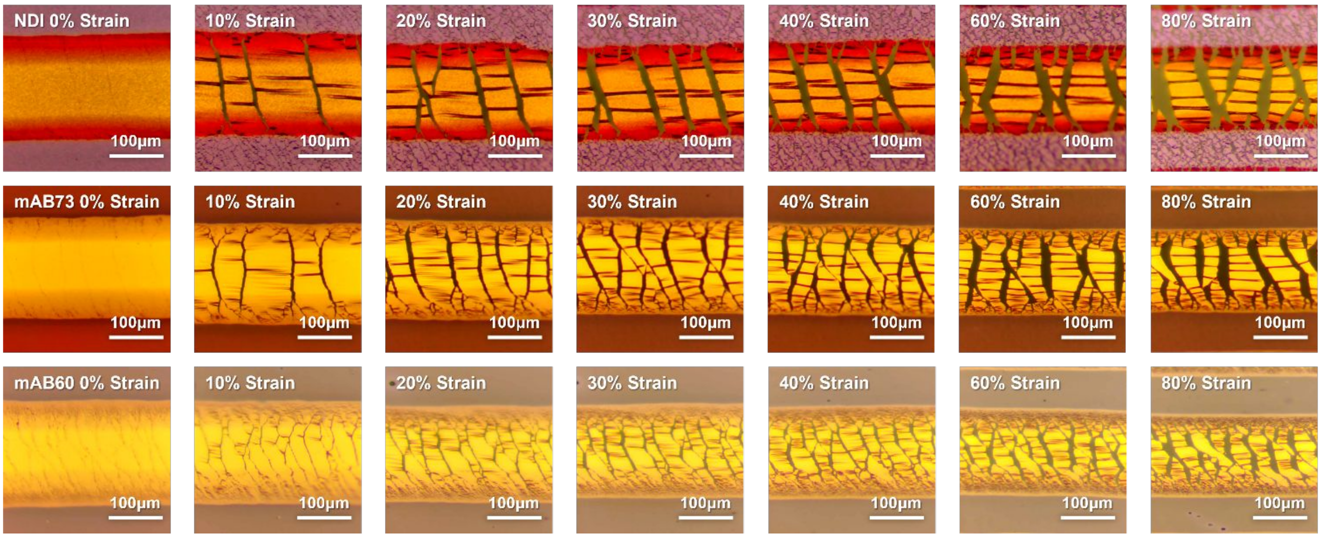

Figure S20. OM images for NDI (top), mAB73 (middle), and mAB60 (bottom) films at different strains with the stretching direction perpendicular to the polymer channel. Note that the perpendicularly stretched gold is 45.3 μm long and 12000 μm wide at 20% strain and 34.3 μm long and 1400 μm wide at 40% strain.

Transfer Line Method (TLM):

At a constant drain voltage (V_d), measure the resistance variation under different channel lengths and gate voltages (V_g). The relationship between the total resistance (R_T), channel resistance (R_{Ch}), and contact resistance (R_c) in the integrated stretchable device is expressed as Equation (1):

$$R_T = R_{Ch} + R_C \tag{1}$$

The relationship of R_{Ch} is derived through the linear region formula of the transfer curve as Equation (2):

$$I_d = \frac{W}{L}\mu_{lin}C\left\{\left(V_g - V_{th}\right)V_d - \frac{1}{2}V_d^2\right\} \tag{2}$$

where I_d is the drain current, V_{th} is the threshold voltage, C is the areal capacitance of the SiO_2 dielectrics, μ_{lin} is the charge mobility in the linear region, and W and L are the channel width and length. Considering the measurement at the linear region, the $V_d^2/2$ term is neglected as the following Equation (3):

$$I_d = \frac{W}{L}\mu_{lin}C\left(V_g - V_{th}\right)V_d \tag{3}$$

R_{Ch} is obtained by dividing V_d by I_d using Ohm's law to form the Equation (4):

$$R_{Ch} = \frac{V_d}{I_d} = \frac{L}{W\mu_{lin}C\left(V_g - V_{th}\right)} \tag{4}$$

As a result, the Equation (4) of R_{Ch} is substituted back into the Equation (1) of R_T. By measuring the total resistance of the transistor and conducting linear regression, the R_c can be obtained as Equation (5):

$$R_T = \frac{L}{W\mu_{lin}C\left(V_g - V_{th}\right)} + R_C \tag{5}$$

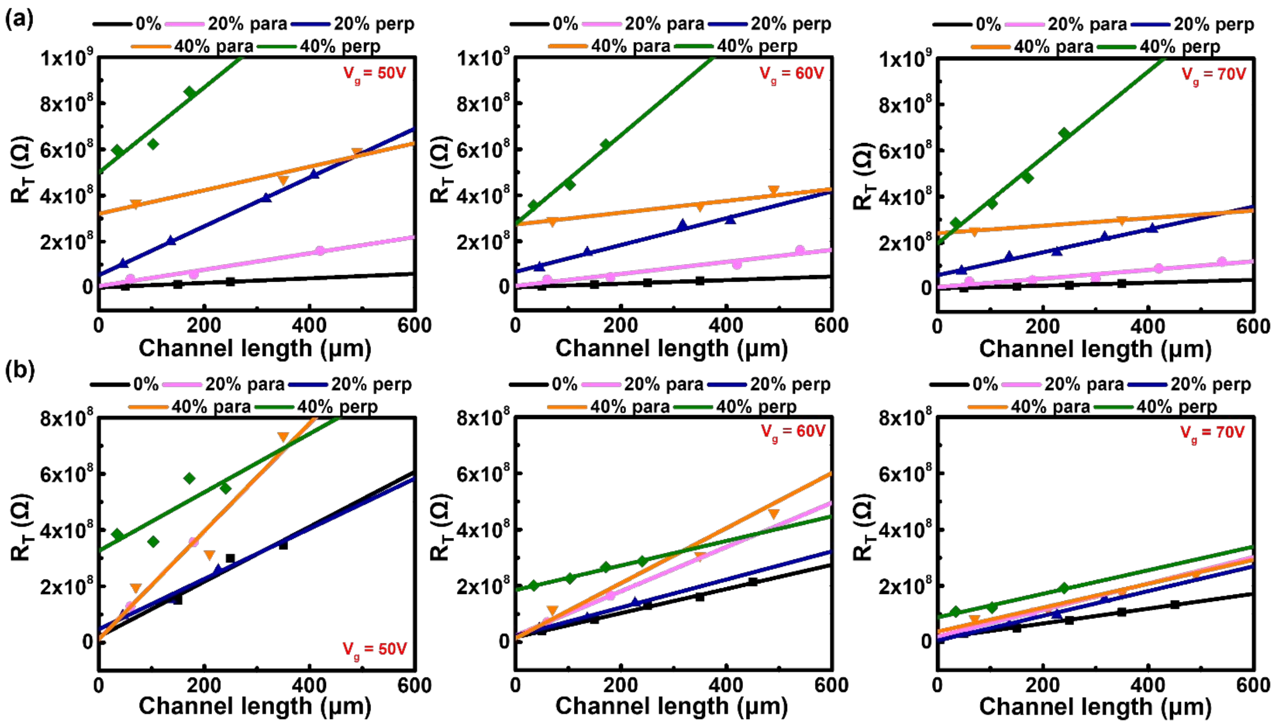

Figure S21. The total resistance (R_T) at V_d = 20 V of (a) NDI (b) and mAB73 OFETs with different channel lengths for extracting the contact resistance. Note that the measurements were conducted at V_g = 50 V (left), 60 V (middle), and 70 V (right) to warrant the linear region characteristics.

Table S7. The contact resistance of NDI and mAB73 with microcracked gold at different strain levels and at V_d = 20 V.

	**NDI**				**mAB73**			
**V_g (V)**	**50**	**60**	**70**	**80**	**50**	**60**	**70**	**80**
**0% (Ω)**	7.7×10^5	6.0×10^5	1.3×10^6	1.5×10^6	2.1×10^7	1.7×10^7	1.4×10^7	1.9×10^7
**20% || (Ω)**	6.9×10^6	6.5×10^6	7.4×10^6	1.5×10^7	1.3×10^7	2.4×10^7	2.0×10^7	9.8×10^6
**20% ⊥ (Ω)**	5.4×10^7	6.8×10^7	5.9×10^7	4.5×10^7	4.7×10^7	2.3×10^7	5.6×10^6	4.8×10^6
**40% || (Ω)**	3.2×10^8	2.7×10^8	2.4×10^8	2.1×10^8	1.2×10^7	1.4×10^7	3.7×10^7	3.1×10^7
**40% ⊥ (Ω)**	5.0×10^8	2.8×10^8	2.0×10^8	2.0×10^8	3.3×10^8	1.9×10	8.8×10^7	4.9×10^7

Table S8. The channel resistance (slope) of NDI and mAB73 with microcracked gold at different strain levels and at V_d = 20 V.

	NDI				mAB73			
V_g (V)	50	60	70	80	50	60	70	80
0% (Ω)	9.9×10^4	7.8×10^4	6.3×10^4	5.6×10^{4y}	9.8×10^5	4.3×10^5	2.6×10^5	1.6×10^5
20% ‖ (Ω)	3.5×10^5	2.6×10^5	1.9×10^5	1.4×10^5	1.9×10^6	7.9×10^5	4.7×10^5	4.2×10^5
20% ⊥ (Ω)	1.1×10^6	5.8×10^5	5.0×10^5	6.1×10^5	8.9×10^5	5.0×10^5	4.4×10^5	3.5×10^5
40% ‖ (Ω)	5.1×10^5	2.6×10^5	1.6×10^5	1.9×10^5	1.9×10^6	9.9×10^5	4.3×10^5	2.8×10^5
40% ⊥ (Ω)	1.8×10^6	1.9×10^6	1.9×10^6	1.4×10^6	1.0×10^6	4.4×10^5	4.2×10^5	3.0×10^5

Table S9. OFET devices parameters, including the electron mobility (μ_e), current contrast (I_{on}/I_{off}), and threshold voltage (V_{th}), of NDI and mAB73 films with microcracked gold at different strain levels. The result was averaged from two different device batches with six units.

		NDI			mAB73		
Strain (%)		μ_e (cm^2 V^{-1} s^{-1})	I_{on}/I_{off}	V_{th} (V)	μ_e (cm^2 V^{-1} s^{-1})	I_{on}/I_{off}	V_{th} (V)
0%		$2.7 \times 10^{-2} \pm 2.1 \times 10^{-2}$	1×10^6	19	$4.2 \times 10^{-3} \pm 1.1 \times 10^{-3}$	6×10^5	15
20%	‖	$1.7 \times 10^{-2} \pm 9.9 \times 10^{-3}$	1×10^3	12	$3.0 \times 10^{-3} \pm 5.5 \times 10^{-4}$	1×10^6	18
	⊥	$2.9 \times 10^{-3} \pm 1.5 \times 10^{-3}$	1×10^4	16	$2.5 \times 10^{-3} \pm 9.0 \times 10^{-4}$	6×10^5	25
40%	‖	$7.8 \times 10^{-3} \pm 4.6 \times 10^{-3}$	1×10^5	10	$3.0 \times 10^{-3} \pm 1.8 \times 10^{-3}$	1×10^6	14
	⊥	$8.7 \times 10^{-4} \pm 4.9 \times 10^{-4}$	2×10^4	16	$1.1 \times 10^{-3} \pm 2.6 \times 10^{-4}$	9×10^5	20

Table S10. OFET devices parameters, including the electron mobility (μ_e), current contrast (I_{on}/I_{off}), and threshold voltage (V_{th}), of stretch-release cycle test for mAB73 films with microcracked gold at a strain level of 40%. The result was averaged from two different device batches with six units.

		mAB73		
**Cycles**		**μ_e (cm^2 V^{-1} s^{-1})**	**I_{on}/I_{off}**	**V_{th} (V)**
**0**		$4.2 \times 10^{-3} \pm 1.1 \times 10^{-3}$	6×10^5	15
**500**	∥	$4.1 \times 10^{-3} \pm 1.1 \times 10^{-3}$	4×10^6	24
	⊥	$5.6 \times 10^{-3} \pm 2.5 \times 10^{-3}$	3×10^5	29
**1000**	∥	$3.1 \times 10^{-3} \pm 1.3 \times 10^{-3}$	1×10^4	25
	⊥	$5.6 \times 10^{-3} \pm 1.8 \times 10^{-3}$	6×10^4	28

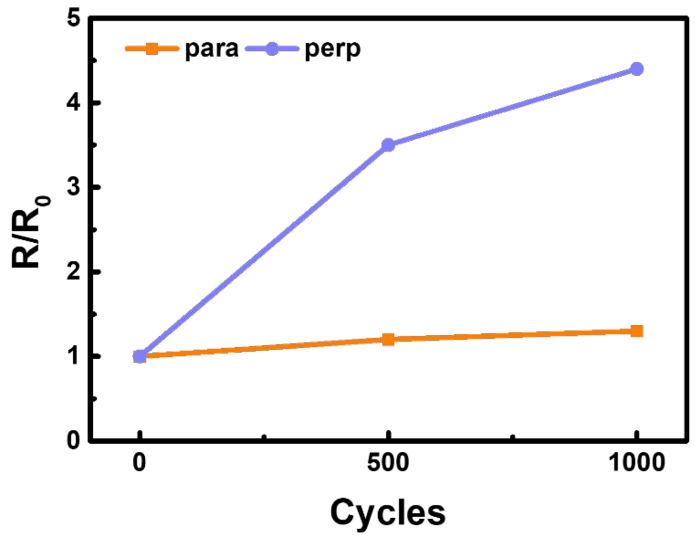

Figure S22. The electrical resistance of electrodes on the polymer films with different stretch–release cycles. Note that the cyclic strain level was 40%.
